# Post-failure resilience and athletes’ training engagement: The chain-mediating roles of perceived meaning in training and value identification

**DOI:** 10.1371/journal.pone.0359579

**Published:** 2026-11-13

**Authors:** Longqiang Chen, Yao Liu

**Affiliations:** 1 Department of Public Physical Education, Fujian Agriculture and Forestry University, Fuzhou, China; 2 College of Basic Education, Wuxi Taihu University, Wuxi, China; Netaji Subhas University of Technology Dwarka, INDIA

## Abstract

Based on theories of sport resilience, meaning construction, and self-determination, this study developed and tested a serial mediation model linking post-failure resilience, perceived meaning in training, value identification, and training engagement. Specifically, the study examined the association between post-failure resilience and training engagement and assessed the serial indirect effect operating through perceived meaning in training and value identification. A final sample of 768 active athletes was recruited from multiple regions and training organizations across China. Data were analyzed using SPSS 26.0 and AMOS 24.0, with descriptive statistics, correlation analysis, confirmatory factor analysis, and structural equation modeling. The results indicated that post-failure resilience was significantly and positively associated with perceived meaning in training, which, in turn, was significantly and positively associated with value identification. Among the focal structural pathways, the association between value identification and training engagement was the strongest. Moreover, the serial indirect effect of post-failure resilience on training engagement through perceived meaning in training and value identification was statistically significant. Multi-group structural equation modeling further revealed no consistent differences in the core structural pathways across sport type, training stage, or gender, suggesting that the proposed pattern of associations was broadly stable across the examined subgroups. These findings provide empirical evidence for the association between post-failure resilience and training engagement in competitive sport and clarify the potential psychological pathways through which this association may operate. The study also provides a theoretical basis and practical implications for structured training management practices, particularly those involving debriefing, feedback processes, and goal alignment.

## Introduction

As the management paradigm in competitive sport increasingly shifts from an exclusive emphasis on performance outcomes toward greater attention to process quality and psychological sustainability, athletes’ capacity to adapt psychologically to setbacks, failure, and negative feedback has become increasingly relevant to training continuity and long-term development. Failure is an inherent feature of competitive sport. Beyond its immediate implications for performance, it may shape athletes’ subsequent judgments and behavioral responses by activating processes of self-evaluation, defensive motivation, and emotional regulation. Existing research suggests that, in training environments characterized by sustained pressure and frequent evaluation, athletes’ cognitive appraisal of failure and their capacity for psychological recovery are closely associated with subsequent training engagement, goal persistence, and psychological exhaustion. These psychological processes may be as consequential as physiological strain or differences in technical competence [1]. Accordingly, post-failure resilience has become an increasingly important topic in sport psychology.

The association between post-failure resilience and training engagement may involve a sequence of interconnected psychological processes. Successful psychological integration of failure experiences may help athletes reconstruct the meaning of training, allowing training to be understood not merely as an instrument for achieving performance outcomes but also as a context for personal growth, competence development, and long-term self-improvement. A stronger sense of meaning in training may, in turn, facilitate value identification by enabling athletes to align training goals more closely with their broader personal value systems. Such alignment may support greater persistence and sustained engagement in training [2,3]. Meaning and value identification are therefore unlikely to remain static. Rather, they may be continuously reconstructed, refined, and consolidated through repeated experiences of challenge, failure, reflection, and adjustment within the training process.

Although sport psychology has examined adaptation and sustained engagement under adversity from the perspectives of resilience, meaning construction, and self-determination, these research traditions have largely developed along parallel theoretical trajectories. Resilience research has primarily addressed athletes’ adaptive capacities, protective resources, and recovery outcomes in response to stress, setbacks, and failure, with particular emphasis on the maintenance or restoration of psychological functioning [4–6]. Research on meaning construction has focused more extensively on how athletes interpret adverse experiences through narrative reconstruction, identity integration, and the restoration of purpose, as well as on the role of meaning in athletic development [7,8]. Self-determination theory, by contrast, has emphasized autonomy support, motivational internalization, self-concordance, and sustained engagement, particularly the process through which externally imposed training demands may become progressively integrated into personally endorsed values and goals [2,9,10].

Collectively, these perspectives illuminate three closely related questions: how athletes recover from adversity, how they reconstruct the meaning of training, and why they continue to invest effort in training. However, the sequential relationship among these processes has not been sufficiently integrated within the specific context of failure in competitive training. The present study therefore brings psychological recovery, meaning reconstruction, and value internalization into a unified structural framework. Post-failure resilience is conceptualized as a psychological resource that enables athletes to regain emotional and cognitive stability following setbacks. Perceived meaning in training refers to athletes’ appraisal of the significance and purpose of continuing to train after failure. Value identification represents the extent to which training goals and demands have been integrated into athletes’ broader systems of personally endorsed values. On this basis, the study examines whether these constructs form a sequential associative structure linking post-failure resilience to training engagement through perceived meaning in training and value identification. Compared with previous research that has examined resilience, meaning, or internalized motivation largely in relation to training outcomes separately, the present study focuses more specifically on how psychological recovery following failure is associated with subsequent meaning reconstruction and value identification. It further evaluates this theoretical integration by testing serial indirect effects and comparing the hypothesized model with alternative structural models.

The Chinese competitive training context provides a practically relevant setting in which to examine this proposed structure. Competitive athletes in China typically participate in systematic and continuous processes of training, evaluation, competition, and stage-based selection. Performance feedback, intra-team competition, and repeated adjustments to training goals are therefore embedded in their long-term developmental experiences. Under such conditions, failure and negative evaluation are not isolated events but recurrent features of the training process. How athletes restore psychological stability after failure, reconstruct the meaning of continued training, and maintain identification with training-related values may therefore be closely associated with their subsequent engagement.

Accordingly, this study develops a serial mediation model comprising post-failure resilience, perceived meaning in training, value identification, and training engagement. Structural equation modeling is used to test the proposed associations and serial indirect effects, while multi-group analyses are conducted to examine whether the core structural pathways vary across relevant contextual characteristics, including sport type and training stage. The study aims to clarify the structural relationships among post-failure resilience, perceived meaning in training, value identification, and training engagement, with particular attention to the serial indirect pathway through perceived meaning in training and value identification. In doing so, it seeks to provide empirical evidence for understanding the psychological processes through which recovery from failure may be associated with sustained training engagement. The findings may also offer theoretical and practical implications for the development of more development-oriented and athlete-centered approaches to training management.

## Theoretical foundations and research hypotheses

### Theoretical foundations

#### Adaptive functions of post-failure recovery capacity (sport resilience theory).

Resilience theory in sport is rooted in developmental psychology and psychopathology, where it was formulated to explain “positive adaptation in adversity.” It conceptualizes resilience as a dynamic process through which individuals achieve functional recovery and adaptive development under conditions of stress, frustration, and threat by mobilizing internal psychological resources alongside external supportive conditions [11,12].

When applied to sport training, failure functions as a normalized and institutionalized stressor. Unattained goals routinely activate salient social-evaluative cues, including intra-team comparisons and coach appraisals, which may elicit shame, frustration, and rumination. These responses deplete attentional resources, strengthen avoidance-oriented tendencies, and ultimately compromise subsequent training quality [13]. In this context, post-failure resilience is conceptually distinct from general stress resistance. It denotes an athlete’s capacity to rapidly reorganize affect and cognition following failure, re-establish perceived control over training goals, and re-enter action sequences oriented toward learning and improvement [4]. Empirical work suggests that this adaptive function operates through three interrelated mechanisms: failure attribution, emotion regulation, and coping behavior. First, resilience recalibrates threat appraisal and attributional patterns, shifting failure interpretations from indicators of deficient competence to diagnostic cues for improvement, thereby sustaining self-efficacy and perceived competence [5]. Second, more effective emotion regulation and attentional control inhibit rumination and affective generalization, reducing the extent to which emotional volatility disrupts training focus [14]. Third, problem-focused coping and feedback-seeking behaviors facilitate the integration of failure episodes into learning trajectories, promoting a functional cycle of failure, correction, and re-engagement [15]. Accordingly, the significance of post-failure resilience extends beyond attenuating negative affect; it preserves the continuity and cumulative nature of training engagement, thereby creating the restart conditions for subsequent psychological transformation and behavioral persistence.

Overall, athletic resilience theory provides a robust framework for explicating psychological regulation in failure contexts. It highlights post-failure recovery capacity as a context-sensitive adaptive mechanism that supports emotional repair, cognitive restructuring, and behavioral re-initiation. By sustaining the continuity and cumulative nature of the training process, this capacity not only enables rapid stabilization following acute setbacks but also furnishes the psychological preconditions for meaning construction and value internalization. Consequently, it functions as a critical initiator of training persistence and long-term developmental trajectories.

#### Integration of failure experiences and the construction of training meaning (meaning-making theory).

Meaning construction theory was first articulated systematically by Park and Folkman (1997) within the stress–coping tradition to account for the psychological process through which individuals appraise, repair, and reconstruct situational meaning when confronted with stressors or major life events [16]. At its core, the framework posits that cognitive processing enables individuals to realign event meanings with pre-existing goals and value systems. Subsequent work refined the architecture of meaning construction by distinguishing two principal pathways—assimilation and accommodation—and by advancing a process model linking situational meaning, global meaning, and meaning outcomes, thereby specifying meaning generation as an ongoing integrative process rather than a discrete reaction [17]. This framework has since been applied across domains including trauma adaptation, health psychology, educational development, and competitive sport to explain how individuals restore purpose, direction, and action legitimacy under adversity.

In competitive training settings, failure constitutes more than a performance setback; it can precipitate identity threat and meaning disruption. This risk is amplified in contexts dominated by performance narratives, where self-worth is tightly coupled to quantifiable outcomes and win–loss records. Under such conditions, failure is more likely to be experienced as a global negation of the self, increasing susceptibility to meaning crises, motivational collapse, and withdrawal tendencies [7,18]. Importantly, integrating failure is not reducible to suppression or emotional rebound. It entails embedding failure episodes within an explanatory account of training development, construing them as diagnostic feedback for skill acquisition, prompts for goal recalibration, or tests of value commitment. This interpretive work restores the comprehensibility of training, re-establishes its motivational purpose, and supports an affectively sustainable narrative for responding to setbacks [17]. Research on meaning and purpose among elite athletes further indicates that adversity often functions as a catalyst for meaning clarification. Although failure may initially destabilize perceived significance, it can also provide a fertile context for deepening purpose and consolidating meaning [8]. When failure experiences are successfully integrated, athletes are more likely to construe training as a route to self-actualization, competence refinement, and value realization. Such construal strengthens the stability and durability of training meaning and, in turn, provides a psychological platform for subsequent value identification and training engagement [16,19].

Overall, meaning construction theory explicates how failure experiences can be transformed from threatening events into developmental material. It underscores that training meaning is not an automatic byproduct of participation but a constructed interpretive achievement that emerges after meaning disruption is cognitively processed, repaired, and narratively integrated. Greater integration of failure experiences increases the likelihood that training will be supported by purposive and value-consistent interpretations. Meaning thus operates as a pivotal psychological conduit linking post-failure resilience to downstream value internalization and sustained training engagement.

#### Value internalization of meaning and training engagement (self-determination theory).

Self-determination theory (SDT), advanced by Deci and Ryan within the motivation research tradition, proposes that behavioral quality hinges on the extent to which regulation shifts from external control toward autonomous motivation. Central to SDT is the claim that autonomy, competence, and relatedness constitute essential psychological conditions for sustained engagement and well-being [9]. Subsequent elaborations differentiated forms of extrinsic motivation along an internalization continuum—external, introjected, identified, and integrated regulation—demonstrating that values and norms can be progressively assimilated into the self-system, thereby reshaping the volitional quality and stability of behavior [10].

In competitive training contexts, the sense of training meaning generated through the integration of failure experiences provides a critical psychological entry point for value internalization. When training is construed as a pathway to self-actualization, competence development, or long-term goals, athletes are more likely to experience training aims as self-endorsed pursuits aligned with personal values. Motivational regulation therefore shifts toward more autonomous forms, expressed in greater volitional coherence and sustained enactment. By contrast, when training meaning is predominantly anchored in external contingencies—rewards and punishments, rankings, or others’ expectations—failure is more likely to activate introjected pressure, self-criticism, and shame. Although commitment may be maintained in the short term, it is often accompanied by heightened emotional exhaustion and motivational instability, thereby undermining the quality and durability of training persistence [10]. The mechanism through which meaning promotes internalization lies in its capacity to furnish self-acceptable reasons for continuing to train. By providing coherent answers to “why persist,” meaning reframes external demands as self-consistent goals embedded within one’s identity narrative, and this reframing is reinforced through basic need satisfaction. Training that is experienced as meaningful is more likely to be enacted as an autonomous choice, to support competence-building experiences, and to consolidate a sense of effectiveness. It may also strengthen relational bonds through support from significant others, jointly sustaining high-quality engagement [20].

Overall, SDT elucidates the motivational logic through which training meaning translates into value identification. By strengthening internalization and satisfying basic psychological needs, meaning shifts training engagement from externally imposed compliance to self-congruent striving. This transformation enhances the stability and sustainability of engagement and provides a motivational basis for behavioral persistence and long-term developmental trajectories.

### Research hypotheses

In competitive training, failure is recurrent and often accompanied by intensive evaluation. Its psychological implications may extend beyond immediate emotional responses to include reassessments of goal attainability, identity stability, and the perceived legitimacy of continued training [5,15]. Post-failure resilience refers to an athlete’s capacity to regain emotional and cognitive stability following setbacks and to redirect attention toward cues for learning, adjustment, and improvement [4]. Meaning-making theory suggests that goal-directed behavior can regain purpose and direction when individuals integrate adverse experiences into their broader developmental narratives and construct personally meaningful interpretations of those experiences [16,17]. Such reconstructed meaning may subsequently facilitate value internalization by enabling externally structured goals to become more closely integrated into the individual’s personal value system, thereby strengthening value identification [9,10]. Stronger value identification, in turn, is associated with more sustained behavioral engagement over time [21]. On the basis of this sequential logic, the following hypotheses are proposed.

**H1: Post-Failure Resilience and Perceived Meaning in Training**

Research on resilience suggests that individuals who are better able to restore emotional stability and regain a sense of control following failure are more likely to interpret setbacks as opportunities for learning and development rather than solely as threats to competence [5]. Meaning-making theory further proposes that assigning developmental significance to adverse experiences is an important condition for reconstructing meaning following disruption [17] Accordingly:

H1a: Post-failure resilience is significantly and positively associated with perceived meaning in training.

The attributional structure of failure and the intensity of social comparison may differ systematically between individual and team sports. In individual sports, performance outcomes are more directly attributable to the athlete, potentially increasing the likelihood that failure will be interpreted as evidence of personal inadequacy or as a threat to athletic identity [22]. Under these conditions, the capacity to recover psychologically from failure may be particularly relevant to the reconstruction of training meaning. Therefore:

H1b: The positive association between post-failure resilience and perceived meaning in training is stronger among athletes in individual sports than among those in team sports.

Training stages also differ in selection pressure, developmental stakes, and opportunity costs. At higher competitive levels and during critical selection periods, failure may carry more substantial consequences for athletes’ future opportunities and developmental trajectories [23]. Psychological recovery from failure may therefore become more closely associated with the ability to maintain or reconstruct a sense of meaning in training. Accordingly:

H1c: The positive association between post-failure resilience and perceived meaning in training is stronger at higher competitive levels or during critical selection stages.

**H2: Perceived Meaning in Training and Value Identification**

Meaning-making theory suggests that the reconstruction of meaning involves aligning behavioral goals with broader beliefs, purposes, and personal values [17]. Self-determination theory further proposes that goals perceived as self-congruent are more likely to be internalized and incorporated into the individual’s self-regulatory system [9,10]. Thus, when athletes perceive continued training as personally meaningful, they may be more likely to identify with the values underlying their training goals. Accordingly:

H2a: Perceived meaning in training is significantly and positively associated with value identification.

Developmental research suggests that value systems remain comparatively more malleable during earlier developmental periods and may therefore be more responsive to contextual cues concerning meaning and purpose [24]. In earlier stages of athletic development, perceived meaning in training may consequently play a stronger role in shaping the internalization of training-related values. Therefore:

H2b: The positive association between perceived meaning in training and value identification is stronger at earlier stages of training.

**H3: Value Identification and Training Engagement**

Value identification reflects the extent to which training-related goals and demands are incorporated into an athlete’s personally endorsed value system. Self-concordant goals are generally associated with greater persistence, sustained effort, and behavioral investment because goal pursuit is experienced as more consistent with the individual’s sense of self [25]. Accordingly:

H3a: Value identification is significantly and positively associated with training engagement.

As athletes progress to higher competitive levels, continued participation increasingly requires sustained self-regulation, long-term commitment, and the capacity to maintain effort despite accumulating demands and opportunity costs [23]. Under such conditions, the extent to which training goals are personally endorsed may become increasingly relevant to sustained engagement. Therefore:

H3b: The positive association between value identification and training engagement is stronger at higher competitive levels or during critical selection stages.

**H4: Serial Indirect Effect**

Integrating meaning-making theory with self-determination theory, post-failure resilience may be associated with training engagement through a sequential psychological process involving the reconstruction of training meaning and the subsequent internalization of training-related values [9,17]. Athletes who recover more effectively from failure may be better able to re-establish the meaning of continued training, which may strengthen their identification with training-related values and, in turn, be associated with greater training engagement. Accordingly:

H4a: Post-failure resilience is significantly and indirectly associated with training engagement through the serial mediation of perceived meaning in training and value identification.

In summary, the hypothesized model tested in this study is presented in Fig 1.

**Fig 1:**
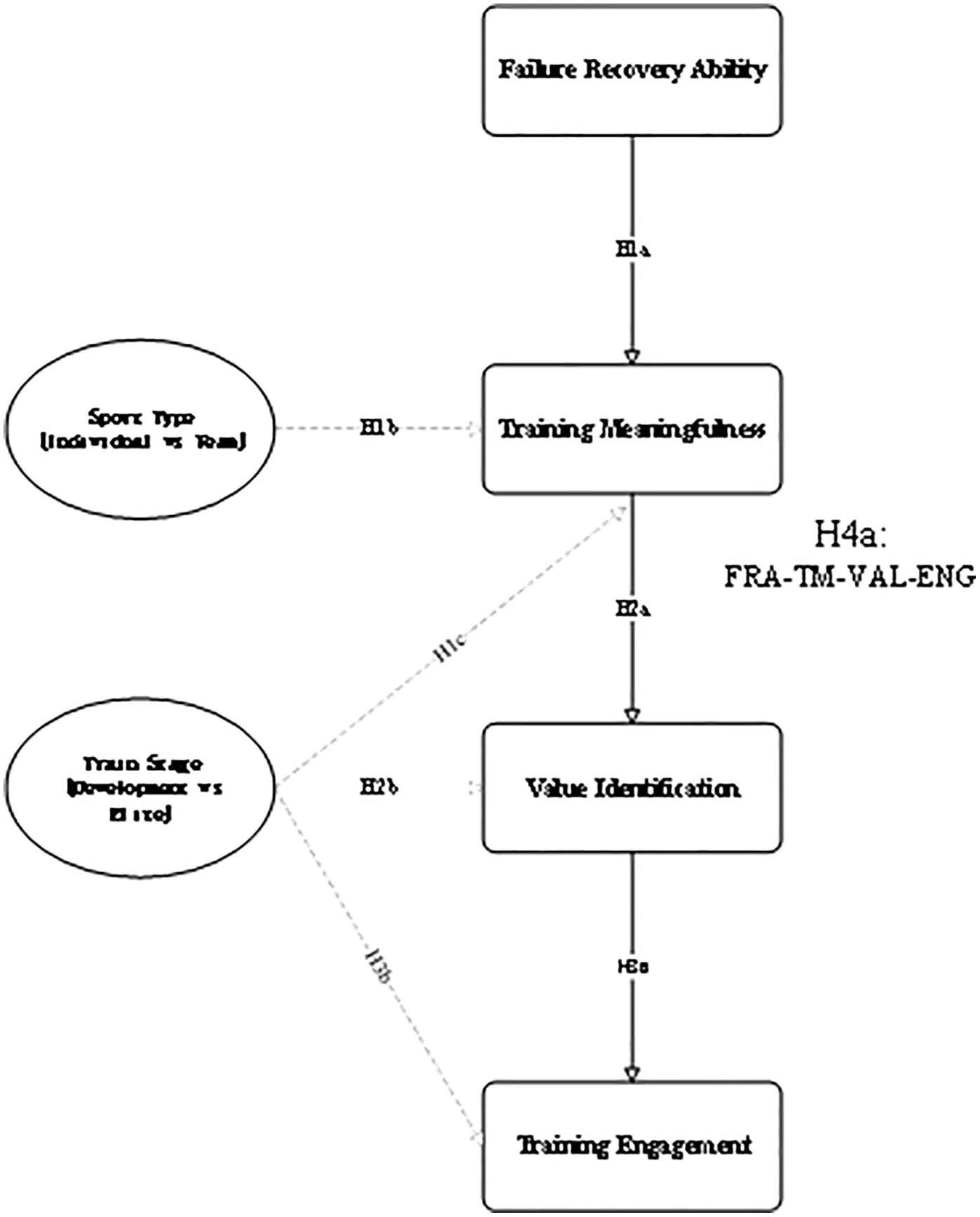
Hypothesized structural model.

## Research design

### Study population and sample sources

This study employed a cross-sectional questionnaire design involving active athletes within China’s competitive training system. Participants were recruited from multiple regions and training organizations across the country. The sample covered North, East, Central, South, and Southwest China and included athletes from 10 provincial-level administrative regions, including Beijing, Jiangsu, Hunan, Hubei, Sichuan, and Guangdong. Participants were drawn primarily from provincial-level professional teams and high-level university athletic teams. The research team recruited eligible athletes through cooperating training institutions and sports teams, and the survey was administered after informed consent had been obtained.

The selection of Chinese competitive athletes was not based on the assumption that the psychological associations examined in this study were unique to the Chinese context. Rather, China’s competitive training system was treated as an empirically relevant setting characterized by structured training arrangements and continuous performance evaluation, thereby providing an appropriate context in which to examine the proposed associative model. Because participants were not recruited through probability sampling from a complete sampling frame with known selection probabilities, individual inclusion probabilities could not be estimated, and sampling weights were therefore not applied. Accordingly, the sample was used primarily to examine the associations among post-failure resilience, perceived meaning in training, value identification, and training engagement, as well as the statistical fit of the hypothesized model. The study was not intended to generate population-representative parameter estimates for Chinese athletes as a whole.

Data were collected through a combination of centralized in-person administration and online follow-up surveys. Participants were recruited between June 18 and August 20, 2025. A total of 860 questionnaires were distributed to athletes, of which 801 were returned. After excluding questionnaires with missing responses, aberrant response patterns, or evidently careless responding, 768 valid questionnaires were retained for analysis, yielding a valid response rate of 89.3%. Before data collection commenced, the study received approval from the Ethics Review Committee of Wuxi Taihu University (Approval No. WXTC-IRB-2025–46). Participation was voluntary, and informed consent was obtained from all adult participants. For participants under 18 years of age, written informed consent was obtained from their parents or legal guardians. All study procedures were conducted in accordance with the Declaration of Helsinki and relevant ethical guidelines, with appropriate safeguards in place to protect participant anonymity, confidentiality, and the responsible use of collected data.

The valid sample comprised athletes aged 16–26 years (M = 20.3, SD = 2.1), including 432 men (56.3%) and 336 women (43.7%). Participants represented multiple sport disciplines, including basketball, volleyball, soccer, track and field, gymnastics, swimming, martial arts, and taekwondo. Based on training task structure, 319 athletes (41.5%) were classified as team-sport participants and 449 (58.5%) as individual-sport participants. Based on the dominant performance demands of their sport, 278 athletes (36.2%) were categorized as endurance-dominant, 301 (39.2%) as skill-dominant, and 189 (24.6%) as strength/competition-dominant. Regarding competitive level, 176 athletes (22.9%) held National Level 1 certification and 352 (45.8%) held National Level 2 certification, whereas 240 (31.3%) were unrated or held local-level ratings. Training experience was concentrated between 4 and 14 years (M = 8.7, SD = 3.2). Based on differences in training phase and selection pressure, participants were grouped into a developmental phase (early-to-mid provincial team/university level) and a critical phase (main-team competition and key selection). The developmental phase included 463 athletes (60.3%), and the critical phase included 305 athletes (39.7%).

### Measurement instruments

Based on the structural pathways specified in the theoretical model, this study operationalized four core latent constructs: post-failure resilience, perceived meaning in training, value identification, and training engagement. A unified self-report questionnaire was developed for athletes to ensure consistency between the operational definitions of the constructs and their theoretical conceptualizations, thereby strengthening the construct validity of the measures. All items were rated on a five-point Likert scale. Depending on item content, response anchors ranged from 1 (strongly disagree or never) to 5 (strongly agree or often). Scores for each construct were calculated as the mean of the corresponding items, with higher scores indicating higher levels of the respective construct. Regarding the sources of the measures, items assessing post-failure resilience and perceived meaning in training were developed primarily on the basis of established theoretical frameworks and relevant research and were contextualized for competitive training. The measure of value identification was adapted from relevant theoretical perspectives and existing measurement content, whereas the measure of training engagement was simplified and contextually revised from an existing sport training scale. Overall, item development and adaptation followed a structured procedure comprising theoretical definition, item generation or contextual adaptation, expert review, athlete feedback, pilot testing, and validation in the formal sample.

The measure of post-failure resilience was developed with reference to the theoretical framework of sport resilience proposed by Fletcher and Sarkar (2012) and the qualitative findings of Galli and Vealey (2008) concerning athletes’ responses to adversity and failure [4,15]. Relevant concepts were translated into items appropriate to competitive training through semantic adaptation and contextual refinement. The resulting six-item measure assessed athletes’ capacity for emotional recovery, cognitive restructuring, and behavioral re-engagement following failure. Example items included: “Even after failing in an important competition or training session, I can quickly regulate my emotions and re-engage in training”; “After a failure, I tend to think about how I can improve rather than repeatedly blaming myself”; and “Setbacks do not have a lasting negative effect on my confidence in my training goals.” In the formal sample, the measure demonstrated satisfactory internal consistency (Cronbach’s α = .847), composite reliability (CR = .848), and convergent validity (AVE = .482). Standardized factor loadings ranged from.643 to.744 and were all statistically significant (p < .001), providing further support for the reliability and convergent validity of the measure.

The measure of perceived meaning in training was developed with reference to Park’s (2010) meaning-making framework and the work of Carless and Douglas (2013) on meaning and narrative processes among elite athletes [7,17]. The five-item measure assessed athletes’ subjective perceptions of the purpose, significance, and developmental value of continued training. Example items included: “Even when I experience setbacks, I still believe that training is important for my growth”; “Training makes me feel that I am working toward meaningful long-term goals”; and “I clearly understand why my continued commitment to training is meaningful.” The measure demonstrated satisfactory internal consistency (Cronbach’s α = .838), composite reliability (CR = .839), and convergent validity (AVE = .512). Standardized factor loadings ranged from.643 to.825 and were all statistically significant (p < .001), further supporting the reliability and convergent validity of the measure.

The measure of value identification was developed with reference to Vallerand’s (1997) hierarchical model of motivation and Sheldon and Elliot’s (1999) self-concordance framework [21,25]. It focused on the extent to which athletes perceived their training goals as personally endorsed and integrated into their broader value systems. The scale comprised six items, including: “My training goals are consistent with the values that I genuinely consider important”; “Continuing to train is consistent with my long-term life plans”; and “Even without external pressure, I would still choose to continue training.” The measure demonstrated high internal consistency (Cronbach’s α = .889), composite reliability (CR = .890), and convergent validity (AVE = .573). Standardized factor loadings ranged from.643 to.825 and were all statistically significant (p < .001), indicating that the measure adequately captured athletes’ identification with the personal value of their training goals.

Training engagement was assessed using core content adapted from the sport training measure developed by Liu et al. (2024) [26], with items simplified and contextually revised for the present study. The seven-item measure assessed sustained effort, attentional concentration, and persistence in training. Example items included: “I exert my full effort during training”; “I remain focused even when training becomes difficult”; and “I rarely miss training sessions without a valid reason.” The measure demonstrated high internal consistency (Cronbach’s α = .915), composite reliability (CR = .915), and convergent validity (AVE = .606). Standardized factor loadings ranged from.643 to.825 and were all statistically significant (p < .001), supporting the reliability and convergent validity of the measure.

Before the formal survey, a pilot test was conducted with 128 athletes. Across the four subscales, Cronbach’s α values exceeded.82, composite reliability values exceeded.85, and AVE values exceeded.53, indicating satisfactory preliminary reliability and convergent validity. The pilot test was also used to evaluate item comprehensibility, semantic clarity, and contextual relevance to competitive training. Several items were subsequently refined in response to athlete feedback. During item development and adaptation, semantic equivalence and conceptual clarity were examined through expert review and athlete consultation. Where items or conceptual expressions were derived from English-language sources, translation and back-translation procedures were used as appropriate. Two researchers with doctoral training in sport psychology independently reviewed the semantic consistency, conceptual correspondence, and contextual appropriateness of the items. Item wording was then further refined on the basis of athlete feedback to minimize potential conceptual distortion arising from translation or contextual adaptation. Because the theoretical boundaries of the four constructs and their corresponding item assignments had been specified a priori on the basis of the theoretical framework, item-development procedures, expert review, and pilot testing, exploratory factor analysis was not used to derive the measurement structure from the formal sample. Instead, the hypothesized four-factor measurement model was directly evaluated using confirmatory factor analysis.

Because all four core constructs were assessed through athlete self-report measures administered at the same time point, procedural remedies were implemented to reduce potential common-method bias. Participation was anonymous and voluntary, and standardized instructions and administration procedures were used to minimize evaluation apprehension and variation in measurement conditions. Before formal data collection, item wording was refined through expert review, athlete feedback, and pilot testing to reduce method effects arising from ambiguous wording, conceptual overlap, or comprehension difficulties. In the subsequent statistical analyses, Harman’s single-factor test and comparison with a single-factor CFA model were additionally used to assess the extent to which common-method variance might threaten the interpretation of the results.

### Data analysis methods

Data processing and statistical analyses were conducted using SPSS 26.0 and AMOS 24.0. Following data collection, data quality screening and preprocessing were performed. Questionnaires were excluded on the basis of missing data, response inconsistency, and aberrant response patterns, resulting in a final analytic sample of 768 athletes. Before structural equation modeling, the distributions of the observed variables were examined to assess their suitability for maximum likelihood estimation. Absolute skewness values were below 1.10, and absolute kurtosis values were below 2.30, indicating no substantial departures from univariate normality. Descriptive statistics and Pearson correlation analyses were subsequently conducted in SPSS to examine the direction and magnitude of the associations among post-failure resilience, perceived meaning in training, value identification, and training engagement, thereby providing preliminary information for subsequent model testing.

At the measurement level, confirmatory factor analysis (CFA) was conducted to evaluate the hypothesized four-factor measurement model. Standardized factor loadings and global model-fit indices were examined to assess the adequacy of the measurement structure. Internal consistency and convergent validity were evaluated using Cronbach’s α, standardized factor loadings, composite reliability (CR), and average variance extracted (AVE). Discriminant validity was assessed using both the Fornell–Larcker criterion and the heterotrait–monotrait ratio (HTMT). According to the Fornell–Larcker criterion, the square root of the AVE for each construct was expected to exceed its correlations with the other constructs. An HTMT value below.85 was adopted as a conservative criterion for discriminant validity. Correlations among the latent constructs were also reported.

To further evaluate the empirical distinctiveness of the four constructs, the hypothesized four-factor model was compared with several competing measurement models. These included a three-factor model in which perceived meaning in training (MEA) and value identification (VAL) were combined; a two-factor model in which post-failure resilience (RES) was combined with MEA and VAL was combined with training engagement (ENG); and a one-factor model in which all items loaded onto a single latent factor. The relative fit of these models was evaluated using χ², degrees of freedom (df), the comparative fit index (CFI), Tucker–Lewis index (TLI), root mean square error of approximation (RMSEA), and standardized root mean square residual (SRMR). Because the four constructs and their item assignments had been specified a priori on the basis of the theoretical framework, item-development procedures, expert review, and pilot testing, exploratory factor analysis was not repeated in the formal sample. Instead, CFA was used directly to test the predefined measurement structure.

Because all four core constructs were assessed through athlete self-report measures administered at the same time point, potential common-method bias was also examined statistically. First, Harman’s single-factor test was conducted by entering all 24 measurement items into an unrotated exploratory factor analysis and examining the proportion of variance accounted for by the first factor. Second, the fit of a single-factor CFA model was compared with that of the theoretically specified four-factor model to determine whether a single latent factor could adequately account for the covariance among the items. These procedures were used as diagnostic assessments of the potential influence of common-method variance and were not interpreted as providing definitive evidence that common-method bias was absent.

At the structural level, once the adequacy of the measurement model had been established, the full serial mediation model was estimated. Six structural paths were specified: RES → MEA, RES → VAL, RES → ENG, MEA → VAL, MEA → ENG, and VAL → ENG. Including both adjacent and nonadjacent paths allowed the indirect effects to be evaluated without inferring mediation solely from the significance of sequential component paths. The fit of the structural model was evaluated separately from that of the measurement model using χ², χ²/df, CFI, TLI, RMSEA with its 90% confidence interval, and SRMR.

Effect decomposition included the direct effect of RES on ENG, two specific simple indirect effects (RES → MEA → ENG and RES → VAL → ENG), the serial indirect effect (RES → MEA → VAL → ENG), the total indirect effect, and the total effect. Both unstandardized coefficients (B) and standardized coefficients (β) were reported. For indirect effects, bootstrap standard errors and bias-corrected 95% confidence intervals were additionally provided. Statistical significance was assessed using a nonparametric bootstrap procedure with 5,000 resamples, with an indirect effect considered statistically supported when its bias-corrected 95% confidence interval did not include zero. The significance of the direct RES → ENG path after inclusion of the mediators was used descriptively to distinguish between partial and full mediation. In addition, coefficients of determination (R²) were reported for the three endogenous latent variables—perceived meaning in training, value identification, and training engagement—to indicate the proportion of variance explained by the structural model.

To examine whether the theoretically specified ordering of the variables provided a comparatively better representation of the observed covariance structure, and to reduce reliance on a single structural specification, several alternative structural models were also tested. The four-factor measurement structure was held constant across all models. The competing models included the hypothesized serial mediation model, a reverse-sequence model, a parallel mediation model, and a direct-effects model. The hypothesized model specified the sequence RES → MEA → VAL → ENG while retaining the direct RES → ENG path. The reverse-sequence model reversed the order of the two mediators to RES → VAL → MEA → ENG while retaining the RES → ENG path. The parallel mediation model specified RES → MEA, RES → VAL, MEA → ENG, and VAL → ENG without specifying a sequential relationship between MEA and VAL. The direct-effects model omitted the mediating pathways involving MEA and VAL.

All competing models were estimated using the same measurement structure and estimation method. Their relative fit was compared using χ²/df, CFI, TLI, RMSEA, SRMR, and relevant information criteria. Because the study employed a cross-sectional design, these alternative-model comparisons were interpreted solely as tests of the relative statistical compatibility of competing theoretical structures with the observed covariance pattern. They were not used to establish temporal ordering or causal direction among the variables.

To examine the stability of the structural relationships across subgroups, multi-group structural equation modeling was conducted. Grouping variables included sport type (team sports, n = 319; individual sports, n = 449), training stage (developmental stage, n = 463; critical stage, n = 305), and gender (male, n = 432; female, n = 336). Before structural path comparisons were undertaken, measurement invariance was evaluated for each grouping variable. A configural invariance model was first estimated to determine whether the same four-factor structure was supported across groups. Metric invariance was then tested by constraining corresponding factor loadings to equality across groups. Structural path comparisons were conducted only after adequate measurement invariance had been established.

Comparisons across configural, metric, and structural constraint models reported χ², df, Δχ², Δdf, and exact p-values for adjacent models. Changes in CFI and RMSEA were also examined when evaluating invariance. Because χ² difference tests are sensitive to sample size, ΔCFI ≤ .010 and ΔRMSEA ≤ .015 were adopted as supplementary criteria for judging whether additional equality constraints produced a meaningful deterioration in model fit. Structural path differences were tested by comparing a model in which the target path was freely estimated across groups with a model in which that path was constrained to equality. A statistically significant deterioration in model fit after imposing the equality constraint was interpreted as evidence that the corresponding path differed across groups.

On the basis of the prespecified hypotheses, the comparison of the RES → MEA path across sport types and the comparisons of the RES → MEA, MEA → VAL, and VAL → ENG paths across training stages were treated as confirmatory tests. Path comparisons across sport types that were not specified a priori, as well as all gender-based path comparisons, were treated as exploratory analyses. To reduce the familywise Type I error rate associated with multiple exploratory comparisons, Holm’s step-down correction was applied to the p-values within each grouping variable. Both unadjusted p-values and multiplicity-adjusted results were reported. Confirmatory and exploratory analyses were clearly distinguished in both the presentation and interpretation of the results.

Individual athletes served as the unit of statistical analysis. Because the final anonymized dataset did not retain identifiers for training organizations or sports teams, the potential clustering of athletes within organizational or team-level units could not be modeled directly. Accordingly, cluster-robust standard errors and multilevel structural equation models were not estimated.

## Research findings

### Descriptive statistics and correlation analysis

To characterize the distributional properties of the core constructs and their bivariate associations, we first conducted descriptive statistics and Pearson correlation analyses for post-failure resilience (RES), perceived meaning in training (MEA), value identification (VAL), and training engagement. (ENG). These analyses provide an empirical basis for subsequent measurement model evaluation and structural path testing.

Table 1 summarizes the descriptive statistics. Mean scores were 3.28 (SD = 0.76) for RES, 3.26 (SD = 0.81) for MEA, and 3.26 (SD = 0.85) for both VAL and ENG. These values indicate meaningful inter-individual variability across key psychological constructs and training-related experiences, providing adequate variance for correlation estimation and model identification.

**Table 1:** Descriptive statistics.

Variable	Mean	Standard Deviation
Post-failure Recovery Ability (RES)	3.28	0.76
Training Meaning (MEA)	3.26	0.81
Value Identification (VAL)	3.26	0.85
Training Engagement (ENG)	3.26	0.85

Fig 2 presents the Pearson correlation matrix for the core study variables. Post-failure resilience (RES) was significantly and positively correlated with perceived meaning in training (MEA; r = .58), value identification (VAL; r = .41), and training engagement (ENG; r = .36). These results indicate that higher levels of post-failure resilience were associated with stronger perceived meaning in training, greater value identification, and higher levels of training engagement. MEA was also significantly and positively correlated with VAL (r = .60) and ENG (r = .44). Among the observed bivariate associations, the correlation between VAL and ENG was the strongest (r = .64), indicating a particularly close positive association between value identification and training engagement. Overall, none of the correlation coefficients reached an excessively high level, suggesting no obvious concern regarding substantial overlap among the constructs at the bivariate level. Moreover, the directions of the observed associations were consistent with the hypothesized relationships, providing preliminary empirical support for subsequent structural model testing.

**Fig 2:**
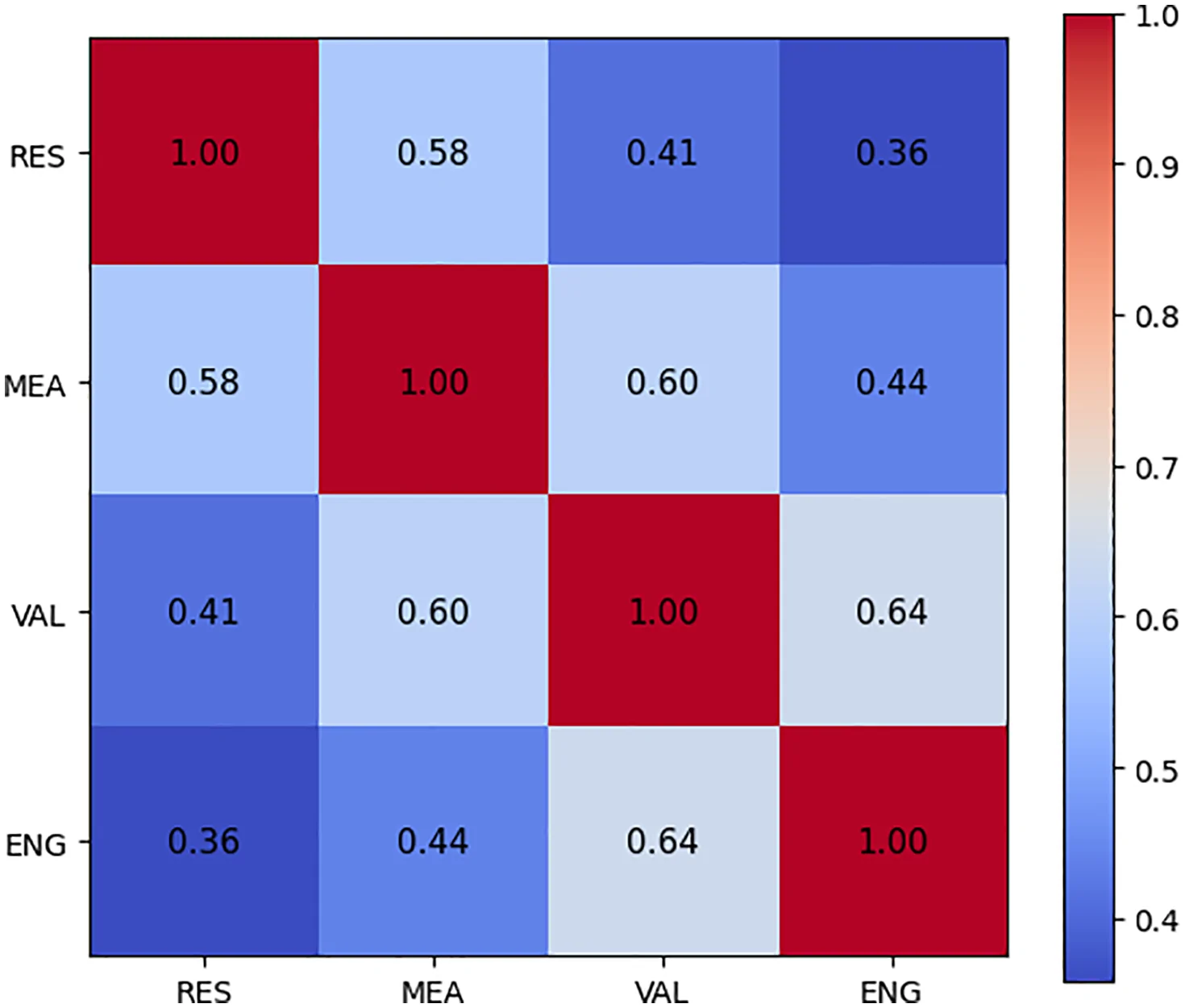
Pearson correlation matrix among core variables.

### Measurement model validation: Confirmatory factor analysis (CFA)

To evaluate the measurement quality and empirical distinctiveness of post-failure resilience (RES), perceived meaning in training (MEA), value identification (VAL), and training engagement (ENG), a four-factor measurement model comprising 24 items was specified and tested using confirmatory factor analysis with maximum likelihood estimation. All items loaded significantly on their intended latent constructs (p < .001), with standardized factor loadings ranging from.643 to.825, indicating that the observed indicators adequately represented their corresponding constructs.

The reliability and convergent validity results for the four constructs are presented in Table 2. Cronbach’s α coefficients for RES, MEA, VAL, and ENG were.847,.838,.889, and.915, respectively, while composite reliability (CR) coefficients were.848,.839,.890, and.915, respectively. All values exceeded the commonly accepted threshold of.70, indicating satisfactory internal consistency. The average variance extracted (AVE) values for MEA, VAL, and ENG were.512,.573, and.606, respectively, all exceeding the recommended threshold of.50. The AVE for RES was.482, slightly below this criterion. However, its CR was.848, and all standardized factor loadings were statistically significant, ranging from.643 to.744. Taken together, these results suggest that the convergent validity of RES was marginal but remained acceptable when considered alongside its satisfactory composite reliability and significant factor loadings.

**Table 2:** Reliability and convergent validity of the constructs.

Construct	Cronbach α	CR	AVE
RES	0.847	0.848	0.482
MEA	0.838	0.839	0.512
VAL	0.889	0.890	0.573
ENG	0.915	0.915	0.606

Discriminant validity was further assessed using the Fornell–Larcker criterion and the heterotrait–monotrait ratio (HTMT). The square roots of the AVE values for RES, MEA, VAL, and ENG were.694,.716,.757, and.779, respectively. Latent construct correlations ranged from.402 to.702: RES–MEA = .660, RES–VAL = .470, RES–ENG = .402, MEA–VAL = .695, MEA–ENG = .493, and VAL–ENG = .702. For each construct, the square root of the AVE exceeded its correlations with the other constructs, thereby satisfying the Fornell–Larcker criterion. The corresponding HTMT values were.669,.468,.403,.690,.490, and.701, respectively, with the highest value observed between VAL and ENG (.701). All HTMT values were below the conservative threshold of.85. Taken together, the Fornell–Larcker and HTMT results provided converging evidence that the four constructs were empirically distinguishable.

To further determine whether the hypothesized four-factor structure provided a better representation of the data than more parsimonious measurement structures, the four-factor model was compared with competing three-factor, two-factor, and one-factor models. In the four-factor model, RES, MEA, VAL, and ENG were specified as distinct but correlated latent constructs. In the three-factor model, MEA and VAL were combined because of their relatively close theoretical relationship. In the two-factor model, RES was combined with MEA, whereas VAL was combined with ENG. In the one-factor model, all 24 items were specified to load onto a single latent construct. The model-comparison results are presented in Table 3.

**Table 3:** Comparison of alternative measurement models.

Model	Factor Specification	χ²/df	CFI	TLI	RMSEA	SRMR
Four-factor model	RES/ MEA/ VAL/ ENG	0.937	1.000	1.002	0.000	0.021
Three-factor model	RES/ MEA + VAL/ ENG	3.419	0.936	0.929	0.056	0.058
Two-factor	RES + MEA/ VAL + ENG	6.811	0.844	0.828	0.087	0.078
One-factor model	All items loaded on one factor	12.161	0.699	0.670	0.121	0.112

The hypothesized four-factor model demonstrated the best overall fit to the data, χ²(246) = 230.546, χ²/df = 0.937, CFI = 1.000, TLI = 1.002, RMSEA = .000, and SRMR = .021. Because the χ² value was slightly lower than the degrees of freedom, CFI reached its upper boundary, and TLI slightly exceeded 1.00, these indices were not interpreted in isolation. Instead, model adequacy was evaluated on the basis of the overall pattern of fit indices, the low SRMR, theoretical interpretability, and comparisons with the competing measurement models. The fit of the three-factor model deteriorated after MEA and VAL were combined, with further deterioration observed for the two-factor model. The one-factor model exhibited the poorest fit. Collectively, these model comparisons provided additional support for conceptualizing RES, MEA, VAL, and ENG as four related but empirically distinguishable latent constructs.

Because all four core constructs were assessed using athlete self-report measures administered at the same time point, potential common-method bias was also examined. Harman’s single-factor test was first conducted by entering all 24 items into an unrotated exploratory factor analysis. Four factors with eigenvalues greater than 1 were extracted, and the first factor accounted for 39.19% of the total variance, below the commonly used reference threshold of 40%. In addition, the one-factor CFA model presented in Table 3 exhibited substantially poorer fit than the theoretically specified four-factor model, indicating that a single latent factor did not adequately account for the covariance structure among the items. Taken together, these results did not suggest that common-method variance constituted a dominant source of covariance in the present data. Nevertheless, these diagnostic procedures cannot completely rule out the possibility of common-method bias.

### Structural path analysis

Building on the supported measurement model, a full serial mediation model was estimated to examine the structural relationships among post-failure resilience (RES), perceived meaning in training (MEA), value identification (VAL), and training engagement (ENG). Six structural paths were specified: RES → MEA, RES → VAL, RES → ENG, MEA → VAL, MEA → ENG, and VAL → ENG. The structural model demonstrated satisfactory overall fit, χ²(246) = 230.546, χ²/df = 0.937, CFI = 1.000, TLI = 1.002, RMSEA = .000, 90% CI [.000,.011], and SRMR = .021. Because the χ² value was slightly lower than the degrees of freedom, CFI reached its upper boundary, and TLI slightly exceeded 1.00, these indices were not interpreted in isolation. Instead, model adequacy was evaluated on the basis of the overall pattern of fit indices and the estimated structural parameters.

The structural path estimates are presented in Fig 3. RES was significantly and positively associated with MEA (B = 0.690, β = .660, p < .001), and MEA was, in turn, significantly and positively associated with VAL (B = 0.751, β = .682, p < .001). VAL was also significantly and positively associated with ENG (B = 0.675, β = .693, p < .001). After accounting simultaneously for the mediators and the remaining structural paths, the direct association between RES and ENG remained positive and statistically significant (B = 0.138, β = .123, p = .005). By contrast, neither the RES → VAL path (B = 0.022, β = .019, p = .703) nor the MEA → ENG path (B = −0.075, β = −.069, p = .264) reached statistical significance. The model explained 43.6% of the variance in MEA, 48.3% in VAL, and 50.2% in ENG (R² = .436,.483, and.502, respectively), indicating that the specified structural relationships accounted for a substantial proportion of variance in the three endogenous constructs.

**Fig 3:**
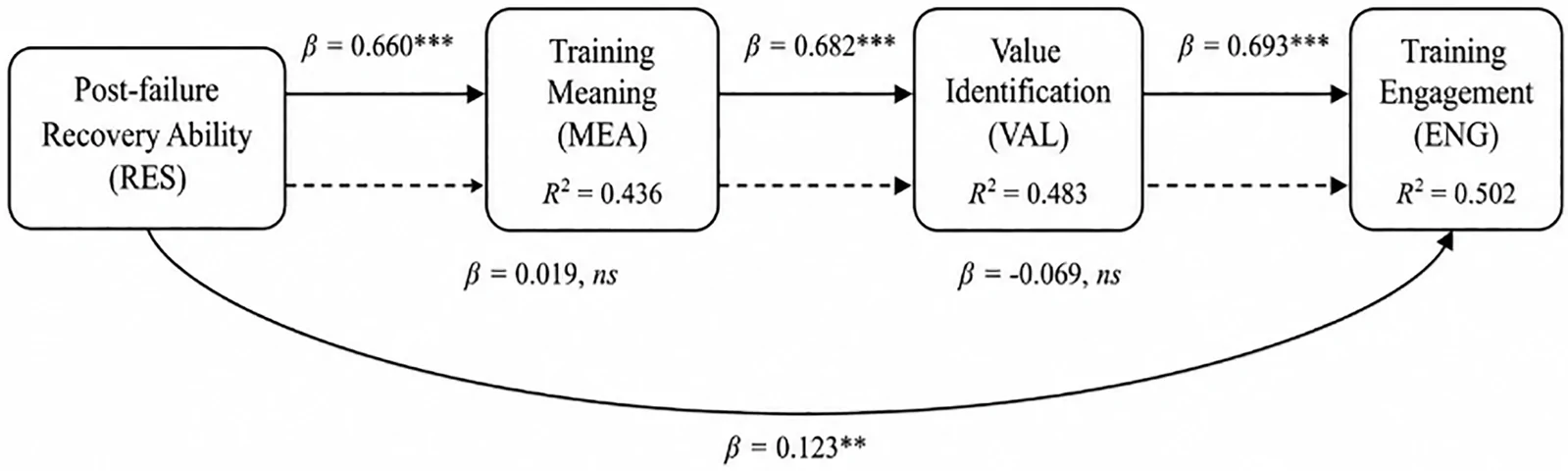
Standardized path coefficients and explained variance in the serial mediation model.

To further examine the indirect association between RES and ENG, the total effect was decomposed into the direct effect, two specific simple indirect effects, the serial indirect effect, and the total indirect effect using a bootstrap procedure. The results are also presented in Table 4. Neither of the two simple indirect pathways was statistically supported. The indirect effect through MEA alone (RES → MEA → ENG) was B = −0.051 and β = −.046, with a bias-corrected 95% confidence interval that included zero. Similarly, the indirect effect through VAL alone (RES → VAL → ENG) was B = 0.015 and β = .013, and its confidence interval also included zero.

**Table 4:** Direct and indirect effects of the serial mediation model.

Effect	B	β	Bootstrap SE	BC 95% CI
Direct effect: RES → ENG	0.138	0.123	0.050	[0.042, 0.234]
RES → MEA → ENG	−0.051	−0.046	0.046	[−0.144, 0.033]
RES → VAL → ENG	0.015	0.013	0.040	[−0.068, 0.091]
RES → MEA → VAL → ENG	0.350	0.312	0.046	[0.266, 0.455]
Total indirect effect	0.313	0.279	0.049	[0.224, 0.412]
Total effect	0.451	0.402	0.045	[0.368, 0.546]

In contrast, the serial indirect pathway RES → MEA → VAL → ENG was statistically significant (B = 0.350, β = .312, 95% BC CI [0.266, 0.455]). This finding indicates that the indirect association between post-failure resilience and training engagement was primarily expressed through a sequential pathway involving perceived meaning in training followed by value identification. The total indirect effect was also statistically significant (B = 0.313, β = .279, 95% BC CI [0.224, 0.412]), as was the total effect (B = 0.451, β = .402, 95% BC CI [0.368, 0.546]). Because the direct RES → ENG effect remained statistically significant after MEA and VAL were included in the model, the results were consistent with partial mediation. Specifically, the serial pathway through perceived meaning in training and value identification accounted for a substantial portion of the association between RES and ENG but did not fully account for this relationship.

To assess whether the theoretically specified ordering of the mediators provided a comparatively better representation of the observed covariance structure, the hypothesized serial model was further compared with three alternative structural models while retaining the same four-factor measurement structure. The theoretically constrained serial model specified the sequence RES → MEA → VAL → ENG while retaining the direct RES → ENG path. This model demonstrated the best overall fit, χ²(248) = 232.066, χ²/df = 0.936, CFI = 1.000, TLI = 1.002, RMSEA = .000, and SRMR = .022.

When the ordering of the two mediators was reversed to RES → VAL → MEA → ENG, model fit deteriorated substantially, χ²(248) = 533.452, χ²/df = 2.151, CFI = .969, TLI = .966, RMSEA = .039, and SRMR = .081. Relative to the hypothesized serial model, the reverse-sequence model yielded ΔAIC = 301.39 and ΔBIC = 301.39. The parallel mediation model, which omitted the MEA → VAL pathway and treated MEA and VAL as parallel mediators, demonstrated acceptable but comparatively weaker fit, χ²(247) = 401.250, χ²/df = 1.624, CFI = .983, TLI = .982, RMSEA = .029, and SRMR = .071, with ΔAIC = 171.18 and ΔBIC = 175.83. The direct-effects model, which omitted the mediating pathways and retained only the direct RES → ENG association, showed further deterioration in fit, χ²(248) = 506.779, χ²/df = 2.043, CFI = .972, TLI = .969, RMSEA = .037, and SRMR = .116, with ΔAIC = 274.71 and ΔBIC = 274.71.

Taken together, the pattern of fit indices and information criteria provided comparatively stronger statistical support for the theoretically specified RES → MEA → VAL → ENG structure. In particular, modeling perceived meaning in training and value identification as sequentially related constructs was more consistent with the observed covariance structure than treating them as parallel mediators or reversing their proposed order. However, given the cross-sectional design, these model comparisons should be interpreted solely as evidence of the relative statistical compatibility of the competing structures with the observed data. They do not establish the temporal ordering of MEA and VAL or the causal direction of the relationships among the study variables.

### Moderating effects analysis

To examine the stability of the core structural relationships across subgroups, multi-group structural equation modeling was conducted across sport type, training stage, and gender. Sport type was classified as team sports (n = 319) or individual sports (n = 449); training stage was classified as developmental (n = 463) or critical (n = 305); and gender was categorized as male (n = 432) or female (n = 336). Before comparing structural paths, configural and metric invariance were sequentially evaluated for each grouping variable. Once metric invariance had been established, equality constraints were imposed on the core structural paths to assess their stability across groups.

For sport type, the configural invariance model demonstrated satisfactory fit, χ²(492) = 450.343, CFI = 1.000, TLI = 1.005, RMSEA = .000, and SRMR = .030. After corresponding factor loadings were constrained to equality across groups, the metric invariance model continued to fit the data well, χ²(512) = 457.699, CFI = 1.000, TLI = 1.006, RMSEA = .000, and SRMR = .030. Relative to the configural model, the change in fit was negligible, Δχ²(20) = 7.356, p = .995, ΔCFI = .000, and ΔRMSEA = .000, supporting metric invariance. When the three core structural paths—RES → MEA, MEA → VAL, and VAL → ENG—were simultaneously constrained to equality across sport types, model fit did not deteriorate significantly, χ²(515) = 463.633, Δχ²(3) = 5.934, p = .115, ΔCFI = .000, and ΔRMSEA = .000. These results indicate that the overall pattern of core structural relationships was broadly stable across team and individual sports.

The training-stage analysis yielded a similar pattern. The configural model demonstrated satisfactory fit, χ²(492) = 488.325, CFI = 1.000, TLI = 1.000, RMSEA = .000, and SRMR = .029. The metric invariance model also showed satisfactory fit, χ²(512) = 508.225, CFI = 1.000, TLI = 1.000, RMSEA = .000, and SRMR = .030. The difference between the two models was nonsignificant, Δχ²(20) = 19.901, p = .464, ΔCFI = .000, and ΔRMSEA = .000, supporting metric invariance across training stages. Further constraining the three core structural paths to equality produced no meaningful deterioration in fit, Δχ²(3) = 0.367, p = .947, ΔCFI = .000, and ΔRMSEA = .000. Thus, no overall structural differences were identified between the developmental and critical stages.

The gender-based analysis likewise supported cross-group stability. The configural model demonstrated satisfactory fit, χ²(492) = 455.791, CFI = 1.000, TLI = 1.004, RMSEA = .000, and SRMR = .029. The metric invariance model also fit the data well, χ²(512) = 473.115, CFI = 1.000, TLI = 1.005, RMSEA = .000, and SRMR = .029. The change in model fit was negligible, Δχ²(20) = 17.324, p = .632, ΔCFI = .000, and ΔRMSEA = .000. When the three core structural paths were simultaneously constrained to equality across gender groups, model fit remained essentially unchanged, Δχ²(3) = 0.568, p = .904, ΔCFI = .000, and ΔRMSEA = .000. These findings suggest that the core structural relationships were broadly comparable between male and female athletes.

Given the support for overall structural stability, each core pathway was subsequently examined individually. The results are presented in Table 5. Contrary to H1b, the RES → MEA path did not differ significantly between team and individual sports (β = .699 vs..636, respectively), Δχ²(1) = 0.984, p = .321. Although the point estimate was slightly higher among team-sport athletes, the between-group difference was not statistically significant and was opposite in direction to the hypothesized stronger association in individual sports. H1b was therefore not supported.

**Table 5:** Multi-group SEM results for core structural paths.

Grouping Variable	Path	Group 1 β	Group 2 β	Δχ² (df = 1)	p
Sport Type	RES → MEA	Team: 0.699	Individual: 0.636	0.984	0.321
Sport Type	MEA → VAL	Team: 0.543	Individual: 0.761	3.960	0.047
Sport Type	VAL → ENG	Team: 0.639	Individual: 0.765	0.971	0.324
Training Stage	RES → MEA	Development: 0.658	Critical: 0.659	0.030	0.862
Training Stage	MEA → VAL	Development: 0.673	Critical: 0.697	0.028	0.868
Training Stage	VAL → ENG	Development: 0.706	Critical: 0.679	0.299	0.585
Gender	RES → MEA	Male: 0.685	Female: 0.620	0.319	0.572
Gender	MEA → VAL	Male: 0.680	Female: 0.688	0.163	0.686
Gender	VAL → ENG	Male: 0.712	Female: 0.665	0.069	0.793

No significant differences were observed across training stages. The RES → MEA association was nearly identical in the developmental and critical stages (β = .658 vs..659), Δχ²(1) = 0.030, p = .862. Similarly, the MEA → VAL pathway did not differ significantly across stages (β = .673 vs..697), Δχ²(1) = 0.028, p = .868, nor did the VAL → ENG pathway (β = .706 vs..679), Δχ²(1) = 0.299, p = .585. Accordingly, H1c, H2b, and H3b were not supported. These findings provide no evidence for the hypothesized training-stage differences in the core structural relationships.

Exploratory analyses were conducted for structural path differences that had not been specified by directional hypotheses. For sport type, the unadjusted comparison of the MEA → VAL pathway reached conventional statistical significance, with a weaker association in team sports than in individual sports (β = .543 vs..761), Δχ²(1) = 3.960, p = .047. However, this difference was no longer statistically significant after Holm adjustment (adjusted p = .093). The VAL → ENG pathway also did not differ significantly across sport types (p = .324). Accordingly, the exploratory analyses did not provide robust evidence of sport-type differences in these pathways. None of the three exploratory gender-based path comparisons reached statistical significance (all ps = .572–.793), and the conclusions remained unchanged following Holm correction. Overall, the exploratory analyses provided no consistent evidence of structural differences by sport type or gender.

Taken together, the results supported the four primary association and indirect-effect hypotheses. Post-failure resilience was significantly and positively associated with perceived meaning in training (β = .660, p < .001), supporting H1a. Perceived meaning in training was significantly and positively associated with value identification (β = .682, p < .001), supporting H2a. Value identification was also significantly and positively associated with training engagement (β = .693, p < .001), supporting H3a. The bootstrap analysis further showed that the serial indirect effect through perceived meaning in training and value identification was statistically significant (B = 0.350, β = .312, 95% BC CI [0.266, 0.455]), supporting H4a. Because the direct RES → ENG effect remained statistically significant after both mediators were included, the findings were consistent with a partial mediation pattern rather than full mediation.

In contrast, H1b, H1c, H2b, and H3b were not supported. Thus, of the eight a priori hypotheses, H1a, H2a, H3a, and H4a received empirical support, whereas the four conditional hypotheses concerning differences by sport type or training stage were not supported.

## Discussion

### Meaning generation through post-failure recovery capacity: How setbacks are reintegrated into training narratives

The results indicate that post-failure resilience was significantly and positively associated with perceived meaning in training, and this association remained broadly stable across the overall sample. This finding suggests that athletes’ capacity to recover emotionally and cognitively reframe failure may be related not only to short-term psychological adjustment but also to how they evaluate the significance and value of continued training.

This finding is broadly consistent with the adaptive patterns identified in international research on sport resilience. Galli and Vealey (2008) showed that athletes often engage in reappraisal, learning, and personal growth when responding to adversity [15] Fletcher and Sarkar (2012) and Sarkar and Fletcher (2014) further emphasized that resilience in sport is closely associated with positive adaptation, the maintenance of psychological functioning, and effective coping under conditions of stress and adversity [4,5]. By contrast, Carless and Douglas (2013) and Oblinger-Peters et al. (2025) focused more explicitly on how athletes interpret adversity and sustain a sense of purpose through narrative and identity processes [7,8]. The present study provides an empirical link between these two lines of research by showing that post-failure resilience is associated not only with general adaptive functioning but also with athletes’ perceptions of the meaning of continued training. In this respect, the findings extend resilience research beyond the question of whether athletes are able to recover by drawing attention to how training may be reinterpreted following failure. However, the observed association should not be interpreted as evidence that resilience necessarily precedes meaning reconstruction. The temporal ordering of these processes requires further examination using longitudinal or prospective designs.

From a meaning-based perspective, failure may disrupt the narrative coherence through which athletes interpret their training experiences. In competitive training, repeated setbacks and evaluative feedback may prompt athletes to reconsider the attainability of their goals, the stability of their athletic identity, and the perceived legitimacy of continued effort. Such experiences may temporarily undermine an established “effort–progress–reward” narrative through which training is understood and justified [7]. When this disruption remains unresolved, training may increasingly be experienced as uncontrollable, difficult to interpret, and psychologically draining, thereby weakening the perceived rationale for continued participation. By contrast, the emotional recovery and cognitive reorganization associated with greater post-failure resilience may help athletes redirect attention toward learning and improvement, regain a sense of control over training goals, and restore coherence to their understanding of the training process. These processes may, in turn, provide favorable psychological conditions for the reconstruction of training meaning.

This interpretation is consistent with the emphasis placed by sport resilience theory on context-sensitive processes of adaptation [5]. Post-failure resilience involves more than the restoration of emotional equilibrium. It also reflects the capacity to reorganize cognitive and emotional responses and re-engage in goal-directed behavior following adversity. Within this framework, greater resilience may reduce the likelihood that failure becomes internalized as definitive evidence of incompetence and may instead facilitate its interpretation as informative and potentially correctable feedback. Such reinterpretation may lessen the extent to which identity threat and repetitive negative thinking consume attentional resources, thereby preserving greater capacity for reflective and meaning-oriented processing. Post-failure resilience can therefore be understood as a psychological resource associated with training continuity, cognitive reorganization, and the maintenance or reconstruction of meaning, rather than simply as the ability to recover emotionally.

Meaning-making theory provides a complementary explanation for the positive association between post-failure resilience and perceived meaning in training. When adverse experiences can be integrated into an individual’s broader developmental narrative and rendered psychologically comprehensible, goal-directed behavior may regain purpose and direction. Greater post-failure resilience may therefore be associated with a more effective process of meaning restoration, allowing setbacks to be interpreted less as events that undermine purpose and more as experiences that can be incorporated into a narrative of learning, skill development, and personal growth. In this way, training may remain embedded within a coherent developmental trajectory despite repeated experiences of failure. This perspective provides a plausible theoretical explanation for why athletes with greater post-failure resilience also report a stronger sense of meaning in training [17]

### The internalization pathway of meaning construction: Theoretical linkages between perceived meaning in training and value identification

The findings revealed a significant positive association between perceived meaning in training and value identification. Athletes who reported a clearer understanding of the purpose and value of training also tended to exhibit stronger value identification and greater self-concordance, suggesting that training goals were more closely integrated into their broader personal value systems. Theoretically, this association may reflect a progression from meaning construction to value integration: when athletes can articulate why training matters, training goals may become more readily aligned with their self-concept, personally endorsed values, and identity-related aspirations.

This finding is broadly consistent with existing research on meaning in sport, identity integration, and motivational internalization. From the perspective of athlete narratives and identity development, Carless and Douglas (2013) argued that when competitive experiences are incorporated into a coherent personal developmental narrative, training and competition can acquire identity-relevant meaning that extends beyond immediate performance outcomes [7]. Oblinger-Peters et al. (2025) further suggested that meaning and purpose in elite sport are not fixed but are continually renegotiated through adversity, transitions, and developmental turning points [8]. At the same time, research grounded in self-determination theory has emphasized autonomy support, self-concordance, and value internalization as important foundations of high-quality motivation [2,3]. Extending these lines of research, the present study distinguishes perceived meaning in training from value identification as two closely related but empirically distinct psychological constructs. The former concerns athletes’ understanding of the purpose, significance, and developmental value of training, whereas the latter reflects the extent to which training goals are experienced as consistent with and integrated into their personal value systems. The strong positive association between the two therefore supports their theoretical proximity without implying conceptual equivalence.

From a theoretical perspective, the association between perceived meaning in training and value identification may first be understood in terms of the internalization of behavioral legitimacy. When training is experienced as congruent with personal growth, skill development, or long-term aspirations, athletes may be better able to maintain the perception that continued training is worthwhile and personally meaningful. Such meaning may reduce the extent to which the perceived value of training depends on short-term fluctuations in performance. Training may therefore retain a coherent rationale even under conditions of frustration, fatigue, or uncertainty. Compared with viewing training solely as an instrument for achieving performance outcomes, athletes with a stronger sense of meaning may be more likely to position training within a broader narrative of self-development. Such narrative integration may, in turn, be associated with more stable identification with training-related values [27].

Self-determination theory provides a complementary explanation for this relationship. The transformation of externally structured goals into more autonomous forms of regulation depends on the internalization of behavioral values, whereby regulation may shift from externally controlled forms toward identified and, potentially, integrated regulation [20]. From this perspective, perceived meaning in training may provide a cognitive and motivational basis for internalization by offering athletes a personally meaningful rationale for continued participation. When training goals are understood as meaningful, they may be experienced less as externally imposed demands driven by rewards, punishment, rankings, or others’ expectations and more as personally endorsed pursuits that are consistent with the athlete’s broader sense of self. As this personally meaningful rationale becomes more coherent and stable, training goals may become more closely aligned with the individual’s existing value system, thereby corresponding with stronger value identification.

Meaning-making theory further clarifies this association at the level of psychological processing. Meaning construction involves the ongoing alignment of situational interpretations with an individual’s broader system of beliefs, goals, and values. Through repeated training experiences, athletes may continually revise and consolidate their understanding of why training matters [19,27]. A stronger sense of meaning in training may therefore reflect a more coherent narrative in which training goals are psychologically connected to self-identity and long-term development. Such coherence may provide favorable conditions for the transition from understanding the significance of training to more fully identifying with its underlying values [25]. In this sense, training may increasingly be experienced as a personally endorsed choice that is congruent with athletes’ self-expectations and long-term life goals.

Nevertheless, the present findings should be interpreted with appropriate caution. The cross-sectional design supports a structural association between perceived meaning in training and value identification but does not establish their temporal ordering. Accordingly, the results cannot be taken as direct evidence that perceived meaning in training precedes value identification or that a process of value internalization has been causally demonstrated.

### The proximal association of value identification: Theoretical linkages between self-concordance and training engagement

The findings revealed a significant positive association between value identification and training engagement, indicating that athletes who perceived their training goals as more fully integrated into their personal value systems also tended to report higher levels of engagement. This finding suggests that value identification is not merely an attitudinal orientation but may represent a form of self-concordance closely related to sustained training behavior. When training is embedded within athletes’ broader understanding of who they are and what they value, continued participation may become more consistent with their self-concept and long-term personal goals, thereby supporting greater persistence, concentration, and behavioral investment.

This finding is broadly consistent with research grounded in self-determination theory on the relationships among value internalization, autonomous regulation, and high-quality engagement. Gagné and Deci (2005) proposed that when individuals identify with behavioral goals and integrate them into their broader value systems, behavioral regulation tends to become more autonomous and persistent [20]. Related research has similarly linked autonomy support and self-concordance with more adaptive motivational and engagement outcomes [2,3]. Liu et al. (2024) further emphasized that training engagement encompasses not only behavioral participation but also sustained effort, attentional concentration, and persistence [26]. Extending these findings, the present study showed that the association between value identification and training engagement was stronger than the direct association between post-failure resilience and training engagement in the full structural model. Moreover, the direct association between perceived meaning in training and training engagement was no longer statistically significant once value identification was included. This pattern suggests that understanding why training is meaningful may not, by itself, be closely associated with higher engagement. Rather, perceived meaning may be more strongly related to engagement when training goals are also experienced as congruent with athletes’ personally endorsed values.

From a theoretical perspective, the positive association between value identification and training engagement may be understood through self-concordance as a mechanism of behavioral regulation. When training goals are experienced as consistent with personally important values, athletes may be more likely to approach training as a self-endorsed activity rather than as an externally imposed demand. Such alignment may be associated with greater persistence, stronger attentional focus, and more stable effort across training situations. Value identification may therefore provide a relatively enduring internal reference point for regulating training behavior, helping to explain why stronger identification with training-related values co-occurs with higher levels of engagement.

Self-determination theory provides a more specific framework for interpreting this association. Integrated regulation represents a highly autonomous form of extrinsic motivation in which behavioral goals are brought into coherence with the individual’s broader values and sense of self [20]. When behavior is regulated in this manner, goal pursuit is more likely to be experienced as self-endorsed and personally meaningful, conditions that are generally associated with greater persistence and high-quality engagement. Within this framework, value identification may reflect a stronger correspondence between training goals and the athlete’s broader sense of self. Training may therefore be experienced not simply as something the athlete wants to do, but as an activity that is consistent with broader aspirations, identity commitments, and long-term development. This interpretation provides a plausible explanation for the strong positive association between value identification and training engagement observed in the present study.

Meaning-making theory offers a complementary explanation for why value identification may be closely associated with training engagement. Perceived meaning in training concerns why continued training is significant, whereas value identification reflects the extent to which that significance has become integrated into the individual’s broader value system. When training goals are incorporated into a more stable framework of personally endorsed values, continued engagement may become less dependent on immediate performance outcomes and more closely aligned with the athlete’s long-term developmental narrative [17]. From this perspective, higher value identification may indicate greater coherence among training goals, self-concept, and long-term aspirations, and this coherence may be closely associated with more persistent and consistent engagement in training.

Nevertheless, this interpretation should remain theoretically bounded. The present study did not directly assess specific forms of motivational regulation, such as identified or integrated regulation. Accordingly, the above explanation represents a theoretically informed interpretation of the observed structural associations rather than direct evidence that a particular process of motivational internalization has been empirically demonstrated.

### Structural stability across groups: Sport type, training stage, and gender

Multi-group structural equation modeling indicated that the core structural pattern linking post-failure resilience, perceived meaning in training, value identification, and training engagement was broadly stable across sport type, training stage, and gender. No consistent between-group differences emerged in the focal structural pathways. Specifically, sport type did not significantly differentiate the RES → MEA association, and the observed coefficients did not follow the hypothesized pattern of a stronger relationship in individual sports. Training stage likewise did not significantly differentiate the RES → MEA, MEA → VAL, or VAL → ENG pathways. Accordingly, H1b, H1c, H2b, and H3b were not supported. Exploratory gender comparisons also revealed no significant structural differences. Overall, the proposed association structure did not appear to vary systematically across the subgroup classifications examined in this study.

This pattern differs to some extent from previous research emphasizing contextual and developmental variation in athletes’ psychological functioning. Existing studies suggest that stress appraisals, coping strategies, and self-regulatory processes vary across individuals and situations, while different stages of athletic development and transition are characterized by distinct demands, stressors, and adaptive resources [23]. Such findings provide a reasonable theoretical basis for considering sport type and training stage as potential boundary conditions. However, the present study found no consistent evidence that these categorical variables altered the strength of the core structural associations. Differences in the intensity of stress, the nature of failure, or the characteristics of the training environment may therefore exist across groups without necessarily producing corresponding differences in the relationships among post-failure resilience, perceived meaning in training, value identification, and training engagement. This distinction is important: group characteristics may influence the level of psychological experience without necessarily moderating the structural relationships among psychological constructs.

The findings for sport type are particularly informative. Team and individual sports differ in patterns of responsibility attribution, social evaluation, goal structure, and interpersonal interaction, which provided the theoretical basis for expecting sport type to influence the association between post-failure resilience and meaning construction. However, the RES → MEA pathway did not differ significantly between team- and individual-sport athletes. Although the exploratory MEA → VAL comparison initially reached conventional statistical significance, the difference was no longer significant after Holm adjustment. The present results therefore do not provide robust evidence that sport type systematically differentiates these pathways. One possible interpretation is that the broad distinction between team and individual sports may not adequately capture the psychological conditions most relevant to meaning construction and value internalization. More proximal contextual characteristics, such as coaching feedback, role stability, performance-evaluation climate, training support, and the social environment surrounding failure experiences, may more closely reflect the conditions actually encountered by athletes. Because these factors were not directly assessed, however, this interpretation should be regarded as a direction for future research rather than as a conclusion established by the present data.

The absence of significant differences across training stages similarly suggests that the hypothesized stage-specific intensification of these relationships should be interpreted cautiously. The original hypotheses assumed that greater opportunity costs, performance pressure, and developmental risk during critical stages might strengthen the relationships linking post-failure resilience, perceived meaning in training, value identification, and training engagement. The observed coefficients, however, were highly similar across developmental and critical stages. This pattern suggests that psychological recovery from failure, the perceived meaning of continued training, and identification with training-related values may retain broadly comparable structural relationships with training engagement across different stages of athletic development. Training stage may influence the frequency or consequences of failure and the intensity of performance pressure, but such differences do not necessarily correspond to systematic changes in the strength of these psychological associations. Given the cross-sectional design, this finding should be interpreted as an absence of evidence for stage-specific structural differences in the present sample rather than as evidence that such developmental changes do not occur.

No significant structural differences were identified across gender groups. Male and female athletes showed comparable directional patterns for the RES → MEA, MEA → VAL, and VAL → ENG pathways, and none of the between-group comparisons reached statistical significance. Thus, the present data provide no evidence of clear gender-specific differences in the associations linking post-failure resilience, perceived meaning in training, value identification, and training engagement. Rather than treating gender as a boundary condition that necessarily alters these psychological relationships, it may be more appropriate to regard it as a background characteristic whose relevance depends on the broader training context. Coaching practices, sport cultures, and evaluation systems may interact with gender in shaping athletes’ experiences, and simple gender-based grouping may therefore be insufficient to capture more nuanced contextual variation.

From a theoretical perspective, the absence of significant contextual differences does not weaken the relevance of the proposed serial model. Instead, the findings suggest that the RES → MEA → VAL → ENG association pattern showed a degree of structural stability across the sport types, training stages, and gender groups represented in the sample. The processes emphasized by meaning-making theory and self-determination theory—namely, the interpretation of experience and the integration of personally endorsed values—may therefore represent relatively general psychological processes within competitive training rather than processes that are necessarily contingent on broad categorical group characteristics [17,20]. Compared with explanations that assume systematic psychological differences solely on the basis of sport type, developmental stage, or demographic characteristics, the present findings point to the potential importance of more proximal training conditions, including coaching feedback, evaluation climate, role stability, and the characteristics of specific failure experiences. Because these variables were not directly examined, however, this interpretation remains a theoretically informed inference derived from the observed absence of stable between-group differences.

## Conclusions and recommendations

### Conclusions

Taking the routine and institutionalized nature of failure experiences in competitive training as its point of departure, this study conceptualized post-failure resilience as a psychological resource that supports stabilization and re-engagement following setbacks. Drawing on meaning-making theory and the perspective of value internalization, the study developed and tested a serial mediation model linking post-failure resilience, perceived meaning in training, value identification, and training engagement. It further examined whether this structural pattern remained stable across different athlete subgroups. The principal conclusions are as follows:

(1) Post-failure resilience was significantly and positively associated with training engagement. Athletes with higher levels of post-failure resilience also tended to report higher levels of training engagement. Theoretically, this association may be related to athletes’ capacity to redirect attention toward opportunities for improvement, restore confidence in the attainability of training goals, and cognitively reframe failure experiences. Training engagement may therefore be associated not only with external training demands but also with athletes’ ability to interpret setbacks in ways that preserve learning, adjustment, and continued participation.
(2) Perceived meaning in training represents an important psychological link between post-failure resilience and training engagement. Post-failure resilience was significantly and positively associated with perceived meaning in training, indicating that athletes who were better able to recover emotionally and cognitively following failure also tended to perceive greater meaning in continued training. From a meaning-making perspective, this association may reflect the extent to which failure experiences can be rendered comprehensible and incorporated into broader narratives of personal growth and athletic development. Such meaning construction may help athletes continue to view training as purposeful and developmentally significant despite setbacks.
(3) Value identification represents a further level of psychological integration through which perceived meaning in training may become connected to athletes’ broader value systems. Perceived meaning in training was significantly and positively associated with value identification, which, in turn, was significantly and positively associated with training engagement. These findings suggest that athletes who more clearly understand the purpose and developmental significance of training also tend to show stronger identification with training-related values. When training goals are experienced as congruent with personally endorsed values and broader self-conceptions, higher levels of training engagement are more likely to co-occur.
(4) A significant serial indirect association was identified between post-failure resilience and training engagement through perceived meaning in training and value identification. Specifically, the RES → MEA → VAL → ENG indirect pathway was statistically significant. The direct RES → ENG association also remained significant after both mediators were included, indicating a partial mediation pattern. These findings are consistent with a sequential associative structure involving perceived meaning in training and value identification. However, because the study employed a cross-sectional design, this serial indirect effect should be interpreted as a statistical pattern of association rather than as evidence of temporal ordering or a confirmed causal mechanism.
(5) Multi-group analyses provided no consistent evidence that sport type, training stage, or gender altered the strength of the core structural pathways. The RES → MEA association did not differ significantly between team and individual sports, and no significant training-stage differences were identified for the RES → MEA, MEA → VAL, or VAL → ENG pathways. Exploratory gender-based comparisons likewise revealed no stable structural differences. Although one exploratory sport-type comparison initially reached conventional statistical significance, the difference was no longer significant after correction for multiple comparisons. Overall, the findings suggest a relatively stable core association structure with limited evidence of subgroup variation. Within the present sample, the RES → MEA → VAL → ENG pattern was broadly comparable across sport types, training stages, and gender groups, although this stability should not be interpreted as evidence that contextual or demographic differences are entirely absent.

### Recommendations

Based on the above findings, the observed association pattern linking post-failure resilience, perceived meaning in training, value identification, and training engagement may provide a useful framework for informing training management. In practice, greater attention could be directed toward institutional arrangements involving communication protocols, feedback processes, and training organization, with failure episodes treated as important opportunities for psychological recovery, meaning reconstruction, and value clarification. However, because the present study employed a cross-sectional design, the following recommendations are derived primarily from statistical associations and theoretically informed interpretations. Their effectiveness as interventions should therefore be evaluated in future longitudinal and experimental research.

(1) Integrate structured post-failure debriefing into routine training communication. Training evaluations, technical corrections, and workload discussions should clearly distinguish between outcome evaluation and information relevant to subsequent improvement. Feedback may be organized around task-specific information, actionable adjustment strategies, and clearly defined next steps, while minimizing humiliating, stigmatizing, or person-focused judgments that may intensify the psychological consequences of failure. Consistency in communication is equally important. Training environments should avoid alternating between messages that encourage learning from errors and practices that stigmatize mistakes. More coherent feedback protocols may provide athletes with clearer contextual support for identifying improvement opportunities, regaining a sense of direction after setbacks, and interpreting failure within a learning-oriented framework.
(2) Consider post-failure resilience as a supplementary indicator of training-process quality rather than as a performance criterion. During key training cycles, coaches and support staff may monitor process indicators such as the pace of emotional recovery following setbacks, the restoration of attentional focus, and athletes’ ability to identify actionable information from failure experiences. Such indicators should be interpreted alongside the quality of coaching feedback, the clarity of training objectives, and the adequacy of post-session debriefing rather than being used as isolated measures of athlete performance. When recovery appears limited, priority may be given to examining whether feedback is sufficiently clear, whether subsequent action plans are feasible, and whether appropriate support structures are available. This approach may be more informative than responding automatically with increased training intensity or punitive measures.
(3) Strengthen the continuity of perceived meaning in training through visible evidence of development and structured reflection. Training goals may be translated into phased and observable indicators of progress so that athletes can recognize developmental gains even when immediate performance outcomes are unfavorable. Periodic reviews, longitudinal training records, and clearly documented progress markers may help sustain a sense of direction across repeated setbacks. Post-failure debriefing may also employ a structured “failure–learning–adjustment” framework that emphasizes interpretation and development rather than evaluative judgment. Such practices may help athletes incorporate failure experiences into a more coherent understanding of their training trajectory and preserve the perceived significance of continued participation.
(4) Incorporate value identification into goal setting and training-related decision-making processes. Opportunities for participatory goal clarification and role discussion may help athletes understand how training demands relate to personally endorsed developmental goals, team responsibilities, and longer-term career aspirations. During the formulation of training plans, stage-specific objectives, and role expectations, coaches may provide greater scope for athletes to express concerns, clarify priorities, and understand the rationale underlying training requirements. Where value identification appears weak, attention may be directed toward unclear goal meaning, ambiguous role expectations, or ineffective communication structures. Goal narratives, explicit role clarification, and transparent evaluation criteria may help strengthen the alignment between training demands and personally meaningful values without equating commitment with passive compliance.
(5) Prioritize a broadly consistent “resilience–meaning–value” support framework while allowing for contextual adaptation. The present study did not identify stable differences in the core structural pathways across sport type, training stage, or gender. Accordingly, the current evidence does not justify fundamentally different intervention frameworks for team and individual sports or for different demographic groups. Practical adjustments may instead be guided by more proximal features of the training environment, including coaching feedback, role stability, evaluation pressure, the characteristics of specific failure experiences, and the availability of training support. In settings characterized by intensive team interaction and public evaluation, particular attention may be directed toward the communication of role-related feedback and social comparison. In contexts where individual responsibility and self-evaluation are more salient, process-oriented feedback, documented evidence of progress, and individualized goal clarification may be especially relevant. These adaptations should be regarded as context-sensitive management strategies rather than as subgroup effects established by the present study.

## Limitations and directions for future research

This study has several limitations. First, the cross-sectional design precludes conclusions regarding the temporal ordering or causal relationships among the study variables. Although the hypothesized serial model demonstrated a comparatively better fit than several alternative structural models, this statistical advantage should not be interpreted as evidence of causal sequencing. Future research should examine these relationships using longitudinal, cross-lagged, or experimental designs.

Second, the core constructs were assessed primarily through athlete self-reports, and the structural model did not systematically incorporate potentially relevant contextual and individual factors, such as competitive level, training experience, coach–athlete interactions, coaching style, and individual psychological characteristics. The robustness of the observed association structure after accounting for a broader range of contextual and individual factors therefore remains to be established. Future studies could strengthen methodological rigor by integrating multi-source data and incorporating more fine-grained indicators of the training environment.

Third, all participants were recruited from China’s competitive training system. This context provides a meaningful setting in which to examine psychological recovery from failure under conditions of systematic training, continuous evaluation, and stage-based selection. However, training organization, coach–athlete relationships, evaluation practices, and sport cultures may differ substantially across countries and regions. The cross-cultural generalizability of the present findings therefore requires further investigation.

Future research could extend the present study in several directions by incorporating multi-source assessments, refining measures of the training context, examining temporal relationships through longitudinal designs, and testing the malleability of the relevant psychological processes through experimental or intervention-based approaches. Replication across athlete samples from different countries and cultural backgrounds would also help clarify the robustness, potential boundary conditions, and broader applicability of the association structure identified in this study.

## Supporting information

S1 Data Fully anonymized dataset underlying the analyses reported in this study. (CSV)

## References

[1] Zhong Z, Jiang H, Wang H. The association of mindfulness with athletes’ fear of failure: The mediating roles of perfectionism and ego-depletion. Front Public Health. 2025;13:1643131. doi: 10.3389/fpubh.2025.1643131 41063962 PMC12500422

[2] Ntoumanis N, Moller AC. Self-determination theory informed research for promoting physical activity: Contributions, debates, and future directions. Psychol Sport Exerc. 2025;80:102879. doi: 10.1016/j.psychsport.2025.102879 40383282

[3] Mossman LH, Slemp GR, Lewis KJ, Colla RH, O’Halloran P. Autonomy support in sport and exercise settings: A systematic review and meta-analysis. Int Rev Sport Exerc Psychol. 2022;17(1):540–63. doi: 10.1080/1750984x.2022.2031252

[4] Fletcher D, Sarkar M. A grounded theory of psychological resilience in Olympic champions. Psychol Sport Exerc. 2012;13(5):669–78. doi: 10.1016/j.psychsport.2012.04.007

[5] Sarkar M, Fletcher D. Psychological resilience in sport performers: A review of stressors and protective factors. J Sports Sci. 2014;32(15):1419–34. doi: 10.1080/02640414.2014.901551 24716648

[6] Gupta S, McCarthy PJ. The sporting resilience model: A systematic review of resilience in sport performers. Front Psychol. 2022;13:1003053. doi: 10.3389/fpsyg.2022.1003053 36619099 PMC9811683

[7] Carless D, Douglas K. “In the boat” but “selling myself short”: Stories, narratives, and identity development in elite sport. Sport Psychol. 2013;27(1):27–39. doi: 10.1123/tsp.27.1.27

[8] Oblinger-Peters V, Henriksen K, Ronkainen NJ. Searching for meaning and purpose in elite sport: A narrative review of sport psychology literature with theoretical insights from psychology. Psychol Sport Exerc. 2025;76:102725. doi: 10.1016/j.psychsport.2024.102725 39222764

[9] Deci EL, Ryan RM. The “what” and “why” of goal pursuits: Human needs and the self-determination of behavior. Psychol Inq. 2000;11(4):227–68. doi: 10.1207/S15327965PLI1104_01

[10] Ryan RM, Deci EL. Self-determination theory: Basic psychological needs in motivation, development, and wellness. Guilford Press; 2017.

[11] Rutter M. Psychosocial resilience and protective mechanisms. Am J Orthopsychiatry. 1987;57(3):316–31. doi: 10.1111/j.1939-0025.1987.tb03541.x 3303954

[12] Masten AS. Ordinary magic. Resilience processes in development. Am Psychol. 2001;56(3):227–38. doi: 10.1037//0003-066x.56.3.227 11315249

[13] Bonanno GA. Loss, trauma, and human resilience: Have we underestimated the human capacity to thrive after extremely aversive events? Am Psychol. 2004;59(1):20–8. doi: 10.1037/0003-066X.59.1.20 14736317

[14] Gucciardi DF, Peeling P, Ducker KJ, Dawson B. When the going gets tough: Mental toughness and its relationship with behavioural perseverance. J Sci Med Sport. 2016;19(1):81–6. doi: 10.1016/j.jsams.2014.12.005 25554654

[15] Galli N, Vealey RS. “Bouncing Back” from adversity: Athletes’ experiences of resilience. Sport Psychol. 2008;22(3):316–35. doi: 10.1123/tsp.22.3.316

[16] Park CL, Folkman S. Meaning in the context of stress and coping. Rev Gen Psychol. 1997;1(2):115–44. doi: 10.1037/1089-2680.1.2.115

[17] Park CL. Making sense of the meaning literature: An integrative review of meaning making and its effects on adjustment to stressful life events. Psychol Bull. 2010;136(2):257–301. doi: 10.1037/a0018301 20192563

[18] Haraldsen HM, Abrahamsen FE, Solstad BE, Halvari H. Narrative tensions in strained junior elite performers’ experiences of becoming elite performers. Front Psychol. 2021;12:645098. doi: 10.3389/fpsyg.2021.645098 34149529 PMC8209255

[19] McAdams DP. The psychology of life stories. Rev Gen Psychol. 2001;5(2):100–22. doi: 10.1037/1089-2680.5.2.100

[20] Gagné M, Deci EL. Self-determination theory and work motivation. J Organ Behav. 2005;26(4):331–62. doi: 10.1002/job.322

[21] Vallerand RJ. Toward a hierarchical model of intrinsic and extrinsic motivation. In: Zanna MP, editor. Advances in experimental social psychology. Vol. 29. Academic Press; 1997. p. 271–360. doi: 10.1016/S0065-2601(08)60019-2

[22] Nicholls AR, Levy AR, Carson F, Thompson MA, Perry JL. The applicability of self-regulation theories in sport: Goal adjustment capacities, stress appraisals, coping, and well-being among athletes. Psychol Sport Exerc. 2016;27:47–55. doi: 10.1016/j.psychsport.2016.07.011

[23] Stambulova N, Alfermann D, Statler T, Côté J. ISSP Position stand: Career development and transitions of athletes. Int J Sport Exerc Psychol. 2009;7(4):395–412. doi: 10.1080/1612197x.2009.9671916

[24] Schwartz SJ, Côté JE, Arnett JJ. Identity development in emerging adulthood: Reviewing the field and looking forward. Dev Psychol. 2012;48(2):155–64.

[25] Sheldon KM, Elliot AJ. Goal striving, need satisfaction, and longitudinal well-being: The self-concordance model. J Pers Soc Psychol. 1999;76(3):482–97. doi: 10.1037//0022-3514.76.3.482 10101878

[26] Liu J-D, Wu J-X, Zou Y-D, Wang Z-H, Zhang S, Hu J-C, et al. Development and initial validation of the Engagement in Athletic Training Scale. Front Psychol. 2024;15:1402065. doi: 10.3389/fpsyg.2024.1402065 39108426 PMC11301410

[27] Habermas T, Bluck S. Getting a life: The emergence of the life story in adolescence. Psychol Bull. 2000;126(5):748–69. doi: 10.1037/0033-2909.126.5.748 10989622

