# Supplementary material for: Post-failure resilience and athletes’ training engagement: The chain-mediating roles of perceived meaning in training and value identification
Source: PLoS One. 2026 Nov 13;21(11):e0359579. doi: 10.1371/journal.pone.0359579 (PMC13766223; doi:10.1371/journal.pone.0359579)
Supplement: Supplementary file 1 [file pone.0359579.s002.docx]

**Review Report**

| **Manuscript ID:** | PONE-D-26-23329 |
| --- | --- |
| **Current title:** | *A Study on the Impact of Resilience Following Failure on Athletes' Training Commitment and Its Chain-Mediated Mechanism* |
| **Recommendation:** | **Major Revision** |

The paper addresses an interesting and relevant issue in sport psychology and has several strengths, including a relatively large sample, a solid theoretical foundation, and an effort to examine subgroup differences. However, several conceptual, methodological, statistical, and reporting concerns currently limit the validity and interpretation of the findings. In particular, the causal language is not appropriate for a cross-sectional self-report design; the mediation model and structural equation model are incompletely reported; several hypotheses are contradicted or unsupported; measurement validation is insufficient; the clustered sampling design is not adequately addressed; and some multi-group significance values appear to be incorrect.

Comment No. 1: Revise the title and causal language to reflect the cross-sectional design.

The current title contains the terms “impact” and “mechanism,” both of which imply causal relationships and temporal ordering. Because all key variables were measured through a cross-sectional survey, the study can establish associations and model-consistent indirect effects, but it cannot demonstrate that post-failure resilience causes training meaningfulness, that meaningfulness causes value identification, or that the variables necessarily operate in the proposed sequence. The title, abstract, hypotheses, results, discussion, and conclusion should therefore avoid expressions such as “predicts,” “promotes,” “translates into,” and “psychological mechanism.” More appropriate wording would include “is associated with,” “shows an indirect association with,” and “is consistent with the proposed serial model.”

As one possible option, the authors may consider the title: “Post-Failure Resilience and Training Commitment Among Chinese Athletes: A Cross-Sectional Serial Mediation Analysis of Training Meaningfulness and Value Identification.” The manuscript should also undergo a careful English-language revision to improve precision, concision, consistency, and readability.

Comment No. 2: Clarify the study design and explain how cluster sampling was handled.

At the beginning of the Methods section, the authors should clearly state that the study used a cross-sectional questionnaire design with multistage stratified cluster sampling, confirmatory factor analysis, structural equation modeling, bootstrap mediation analysis, and multi-group SEM. The sampling subsection should report the number of provinces, institutions, teams, and participants within each cluster, explain how participants were selected, and state whether selection probabilities or sampling weights varied.

Because participants were recruited from teams and institutions, the assumption of independent observations may have been violated. Cluster-robust standard errors, complex-sample procedures, or multilevel SEM should be used where possible. If this is not feasible, the resulting dependence should be acknowledged clearly as a limitation.

Comment No. 3: Provide complete reporting of the mediation and structural equation models.

The manuscript states that the serial indirect effect is significant, but the Results section does not present a complete mediation table. The authors should report the total effect, the direct effect after including the mediators, the specific indirect effects, and the serial indirect effect through training meaningfulness and value identification. Standardized and unstandardized estimates, bootstrap standard errors, 95% bias-corrected confidence intervals, and the proportion of explained variance (R²) should also be provided.

The structural-model fit statistics should be reported separately from the CFA fit statistics and should include χ², degrees of freedom, the exact p-value, χ²/df, CFI, TLI, RMSEA with its 90% confidence interval, and SRMR. The manuscript should clarify whether the direct effect of resilience on training commitment was estimated and whether the model represents full or partial mediation. Figure 3 should be revised accordingly or supported by a complete statistical results table.

Comment No. 4: Resolve inconsistencies between the hypotheses and the reported findings.

Several hypotheses are contradicted or unsupported and should be reported transparently. H1b predicts that the resilience-to-meaningfulness relationship will be stronger in individual sports, whereas the reported coefficients indicate a stronger relationship in team sports; therefore, H1b is not supported. H1c, H2b, and H3b concern training-stage differences, but no significant differences across training stages are reported; therefore, these hypotheses are also not supported. H4a cannot be evaluated fully until the indirect-effect estimates and their confidence intervals are presented.

The authors should add a concise hypothesis-testing table in the Results section showing each hypothesis, the predicted relationship, the corresponding estimate, the significance result, and whether the hypothesis is supported. Any post hoc interpretation, particularly regarding stronger effects in team sports, should be identified explicitly as exploratory.

Comment No. 5: Strengthen measurement validation and address common-method bias.

The four focal measures appear to have been adapted or developed specifically for this study. In the Measurement Instruments section, the authors should state whether validated Chinese versions were available, which items were adopted, modified, or newly written, whether reverse-coded items were used, how translation and back-translation were conducted, whether the pilot sample overlapped with the main sample, and whether exploratory factor analysis was performed before CFA.

Cronbach’s alpha, composite reliability, and AVE are not sufficient to establish measurement quality. The authors should also report item-level standardized loadings, factor correlations, Fornell–Larcker results, HTMT ratios, and fit comparisons for one-, two-, and four-factor models. Because all constructs were measured through the same self-report questionnaire at one time point, common-method variance is an important concern. The manuscript should describe the procedural safeguards used and apply an appropriate statistical assessment.

Comment No. 6: Correct the multi-group SEM reporting and significance values.

The manuscript states that configural and metric invariance were established, but the supporting evidence is not presented. For sport type, gender, and training stage, the authors should report fit indices for configural, metric, and structural invariance models, together with ΔCFI, ΔRMSEA, Δχ², degrees of freedom, and exact p-values.

The reported significance levels in Table 4 also require correction. For one degree of freedom, Δχ² = 5.35 corresponds to approximately p = .021, Δχ² = 9.71 corresponds to approximately p = .0018, and Δχ² = 5.28 corresponds to approximately p = .022. These values should be corrected in Table 4 and in the related narrative. The authors should also clarify whether the subgroup analyses were confirmatory or exploratory and discuss the implications of multiple testing.

Comment No. 7: Moderate the interpretation, practical recommendations, and conclusion.

The discussion currently presents the proposed sequence as an established psychological process, but cross-sectional observational evidence cannot confirm temporal ordering or causality. The authors should test plausible alternative models, including reverse, parallel, and direct-effect models, and report the comparison in the Results section. A better fit of the proposed model would improve its empirical plausibility, although it would still not establish causality.

The practical recommendations should be reframed as theory-guided suggestions for future intervention research rather than as demonstrated consequences of the present data. A dedicated Limitations subsection should discuss the cross-sectional design, self-report bias, common-method variance, possible cluster dependence, measurement adaptation, limited national generalizability, uncontrolled confounders, unequal subgroup sizes, and the absence of longitudinal, coach-rated, behavioral, or objective training data. The conclusion should be revised to correspond strictly to the observed associations.

**The authors are advised to address the following questions thoroughly and provide clear, logical, and well-supported responses.**

**Q1. Model ordering:** In the Data Analysis Methods subsection, specify the reverse, parallel, and direct-effect models to be tested. In the Results section, add a model-comparison table showing the fit of these alternatives against the proposed serial model. What empirical evidence supports the ordering resilience → training meaningfulness → value identification → training commitment?

**Q2. Cluster sampling:** In the Study Population and Sample Sources subsection, report the exact number of provinces, institutions, teams, and participants within each cluster. In the Data Analysis Methods subsection, explain how within-cluster dependence was handled. If clustering was not considered, re-estimate the model using cluster-robust or multilevel methods, or provide a clear explanation in the Limitations section.

**Q3. Complete SEM reporting:** In the Results section, insert one table for mediation analysis and another for multi-group invariance analysis. These tables should report the direct, total, and specific indirect effects; bootstrap confidence intervals; model-fit statistics; invariance statistics; Δχ² values; and exact p-values. Figure 3 should also be revised to show the direct effect, explained variance, and complete model structure, or it should be accompanied by a detailed statistical table.

Although the manuscript addresses a useful research question and includes a potentially valuable dataset, the current version requires major revision because the analytical reporting is incomplete, several hypotheses are unsupported, measurement and sampling concerns remain unresolved, and the conclusions extend beyond what can be justified by a cross-sectional self-report design. A careful major revision would strengthen the paper’s methodological rigor, interpretive balance, and practical significance. The recommended revisions are also expected to enhance the overall quality of the manuscript and increase its potential impact on future research.
