# Supplementary material for: Post-failure resilience and athletes’ training engagement: The chain-mediating roles of perceived meaning in training and value identification
Source: PLoS One. 2026 Nov 13;21(11):e0359579. doi: 10.1371/journal.pone.0359579 (PMC13766223; doi:10.1371/journal.pone.0359579)
Supplement: Supplementary file 2 [file pone.0359579.s004.docx]

**SECOND-ROUND REVIEW REPORT**

**Reviewer 1**

**Manuscript:** Post-failure resilience and athletes’ training engagement: the chain-mediating roles of perceived meaning in training and value identification

**Manuscript No.:** PONE-D-26-23329R1

# Overall Assessment

I have carefully examined the revised manuscript together with the authors’ detailed point-by-point responses to my previous comments. The authors have responded constructively and have made substantial revisions addressing the principal methodological, statistical, and interpretive concerns raised during the first round of review.

The revised manuscript is considerably improved in its treatment of the cross-sectional study design, causal interpretation, sampling and clustering limitations, structural equation modeling, indirect-effect reporting, hypothesis evaluation, measurement validation, multi-group comparisons, common-method bias, alternative structural models, limitations, and practical implications.

In my assessment, the major concerns raised in the previous review have been satisfactorily addressed. Some recommendations concerning presentation were implemented in an equivalent rather than identical format; however, the necessary information is now provided clearly and adequately. I do not consider these differences to require another substantive revision.

My point-by-point assessment is provided below.

# 1. Cross-sectional design and causal language

**Previous concern.** The original manuscript contained terminology that could imply causal relationships or an established psychological mechanism despite the use of a cross-sectional self-report design. I requested that the title, abstract, results, discussion, and conclusion be reframed using appropriately associational terminology.

**Assessment of the revision.** This issue has been satisfactorily addressed. The revised manuscript now consistently distinguishes statistical associations from causal or temporal relationships. The authors appropriately use expressions such as “association,” “indirect association,” “serial indirect effect,” and “relative compatibility” of structural models. They also explicitly acknowledge that model comparison using cross-sectional data cannot establish temporal precedence or causal direction.

The interpretation of the findings is therefore substantially more appropriate for the study design.

**Assessment: Satisfactory.**

# 2. Study design, recruitment, and clustering

**Previous concern.** I requested clarification of the recruitment procedure, sampling structure, selection probabilities, and possible non-independence arising from athletes being recruited from teams and institutions.

**Assessment of the revision.** The revised manuscript now describes the study more accurately as a cross-sectional questionnaire study using multi-region and multi-training-organization recruitment rather than overstating the design as formal probability-based multistage cluster sampling.

The authors report recruitment across 10 provincial-level administrative regions and clarify that a complete probability sampling frame, known individual selection probabilities, and sampling weights were not available.

They also explain that institution- and team-level identifiers were not retained in the final anonymized dataset. Consequently, valid cluster-robust or multilevel re-estimation could not be performed during revision. Importantly, this limitation is now stated explicitly, the individual athlete is identified as the unit of analysis, and the scope of generalization has been appropriately restricted.

This is a transparent and methodologically reasonable response to the available data.

**Assessment: Satisfactory.**

# 3. Structural equation modeling and mediation analysis

**Previous concern.** I requested complete reporting of the structural model, including the direct effect, specific indirect effects, serial indirect effect, total indirect effect, total effect, standardized and unstandardized estimates, bootstrap confidence intervals, model-fit indices, explained variance, and clarification of whether mediation was full or partial.

**Assessment of the revision.** The authors have substantially improved this part of the manuscript.

The revised structural model estimates the relevant direct and indirect pathways simultaneously. The structural-model fit indices are reported separately from the measurement-model results, and the revised mediation analysis provides the direct effect, two specific indirect effects, the serial indirect effect, total indirect effect, and total effect.

Both standardized and unstandardized coefficients are reported, together with bootstrap standard errors and bias-corrected 95% confidence intervals. The manuscript also reports the explained variance for the endogenous constructs.

Because the direct association between post-failure resilience and training engagement remains statistically significant after inclusion of the mediators, the authors now correctly characterize the pattern as partial rather than full mediation.

The revised presentation is substantially more complete and transparent.

**Assessment: Satisfactory.**

# 4. Hypotheses and consistency with the statistical findings

**Previous concern.** Several hypotheses were not supported by the reported results, and I requested that supported and unsupported hypotheses be reported transparently and that exploratory subgroup findings be distinguished from confirmatory hypotheses.

**Assessment of the revision.** The revised manuscript now clearly identifies H1a, H2a, H3a, and H4a as supported, while H1b, H1c, H2b, and H3b are explicitly reported as unsupported.

In particular, H1b is no longer interpreted as supported because the between-group comparison was nonsignificant and the observed point estimates did not follow the hypothesized direction.

The authors also distinguish pre-specified confirmatory comparisons from exploratory subgroup analyses and apply Holm correction to the exploratory multiple comparisons.

Although a separate hypothesis-status table was not added, the relevant evidence is clearly presented in the revised Results and associated tables. I consider this an acceptable presentation.

**Assessment: Satisfactory.**

# 5. Measurement validation and common-method bias

**Previous concern.** I requested greater transparency regarding the development and adaptation of the measures, translation procedures, pilot testing, factor structure, discriminant validity, item-level loadings, competing measurement models, and common-method variance.

**Assessment of the revision.** The measurement section has been substantially strengthened.

The authors now distinguish more clearly between measures developed for the present competitive-training context and measures adapted from previous theoretical or measurement frameworks. The manuscript describes the pilot testing of 128 athletes, expert semantic review, athlete feedback, and translation/back-translation procedures where applicable.

The revised validation includes standardized factor loadings, latent-factor correlations, Fornell-Larcker assessment, HTMT ratios, and comparisons among competing measurement models. Supplementary Table S1 additionally reports item-level standardized factor loadings.

The authors have also expanded their treatment of possible common-method bias through both procedural safeguards and statistical diagnostics. Importantly, they explicitly acknowledge that these procedures cannot completely eliminate the possibility of common-method variance.

These revisions satisfactorily address the major measurement concerns raised previously.

**Assessment: Satisfactory.**

# 6. Multi-group SEM and invariance testing

**Previous concern.** I requested more complete reporting of measurement invariance, corrected significance values, structural-path comparisons, exact model-comparison statistics, and consideration of multiple testing.

**Assessment of the revision.** The authors have re-examined and substantially improved the multi-group SEM analysis.

The revised manuscript reports configural and metric invariance analyses for sport type, training stage, and gender, followed by structural-path equality tests. The manuscript provides χ², degrees of freedom, Δχ², exact p-values, ΔCFI, and ΔRMSEA for the relevant model comparisons.

Table 5 reports the group-specific standardized path coefficients and the corresponding path-comparison statistics.

The previously problematic significance values have been replaced by rechecked analyses. The distinction between confirmatory and exploratory subgroup analyses is now explicit, and Holm adjustment is used for exploratory multiple comparisons.

The revised analysis is much clearer and statistically more defensible.

**Assessment: Satisfactory.**

# 7. Alternative models, interpretation, practical implications, and limitations

**Previous concern.** I requested comparison of plausible alternative structural models, more cautious interpretation of the serial model, moderation of the practical implications, and a dedicated discussion of the study's limitations.

**Assessment of the revision.** The authors have responded appropriately.

The proposed serial structure is now compared with reverse-order, parallel-mediator, and direct-effect alternatives while retaining the same measurement structure. The theoretically proposed serial model demonstrates comparatively better fit than the tested alternatives.

Most importantly, the authors correctly acknowledge that better fit does not establish the true temporal sequence or causal direction of the variables.

The practical implications have also been reframed as theory- and association-informed suggestions requiring future longitudinal or experimental validation rather than as demonstrated intervention effects.

The manuscript now contains a dedicated limitations discussion addressing the cross-sectional design, reliance on self-report data, relevant unmeasured contextual and individual factors, limitations on generalizability, and the need for longitudinal, experimental, multi-source, and cross-cultural research.

These changes substantially improve the balance and credibility of the Discussion and Conclusion.

**Assessment: Satisfactory.**

# Additional Questions Raised in the Previous Review

## Q1. Model ordering

The authors now specify and compare reverse-order, parallel-mediator, and direct-effect alternatives with the proposed serial model.

The proposed RES → MEA → VAL → ENG structure shows comparatively stronger statistical compatibility with the observed covariance pattern than the tested alternatives. The authors appropriately emphasize that this evidence is comparative rather than causal.

Although the model comparisons were presented consecutively in the Results rather than in a separate additional table, the relevant fit indices and information criteria are available.

**Assessment: Satisfactory. No further revision required.**

## Q2. Recruitment clusters

The authors now provide the recruitment information that can be verified and explain why cluster-level re-estimation cannot be undertaken from the anonymized analytic dataset.

They appropriately avoid attempting to reconstruct unavailable cluster identifiers from incomplete information and explicitly acknowledge the resulting limitation.

**Assessment: Satisfactory. No further revision required.**

## Q3. Complete SEM and invariance reporting

The revised manuscript contains a complete effect-decomposition table, reports the explained variance of the endogenous constructs, retains the direct RES → ENG pathway in the model, and provides detailed multi-group invariance and structural-comparison statistics.

Although a separate additional invariance table was not included, the relevant statistics are clearly reported in the Results and Table 5. I consider the presentation sufficiently complete for evaluation and replication.

**Assessment: Satisfactory. No further revision required.**

# Final Assessment

The authors have taken the previous review seriously and have made substantial, technically meaningful revisions. The manuscript is now more transparent regarding its evidential limits, considerably stronger in its SEM and mediation reporting, clearer in distinguishing supported from unsupported hypotheses, more rigorous in measurement validation and subgroup analysis, and more cautious in interpreting cross-sectional findings.

In my assessment, the substantive concerns raised in my first-round review have been satisfactorily resolved. I have no further major or minor scientific comments requiring another revision.

**The revised manuscript is satisfactory, and I support its acceptance for publication in PLOS ONE.**

**Reviewer 1**
