# Supplementary material for: Post-failure resilience and athletes’ training engagement: The chain-mediating roles of perceived meaning in training and value identification
Source: PLoS One. 2026 Nov 13;21(11):e0359579. doi: 10.1371/journal.pone.0359579 (PMC13766223; doi:10.1371/journal.pone.0359579)
Supplement: Peer review history

# Peer review: Decision Letter 0

**DOI:** 10.1371/journal.pone.0359579.r001

**Authors:** Vineet Kumar

2 Jul 2026

PONE-D-26-23329A Study on the Impact of Resilience Following Failure on Athletes' Training Commitment and Its Chain-Mediated MechanismPLOS One

Dear Dr. Liu,

Thank you for submitting your manuscript to PLOS ONE. After careful consideration, we feel that it has merit but does not fully meet PLOS ONE’s publication criteria as it currently stands. Therefore, we invite you to submit a revised version of the manuscript that addresses the points raised during the review process.

Please submit your revised manuscript by Aug 31 2026 11:59PM. If you will need more time than this to complete your revisions, please reply to this message or contact the journal office at plosone@plos.org. When you're ready to submit your revision, log on to https://www.editorialmanager.com/pone/ and select the 'Submissions Needing Revision' folder to locate your manuscript file.

Please include the following items when submitting your revised manuscript:

- A letter that responds to each point raised by the academic editor and reviewer(s). You should upload this letter as a separate file labeled 'Response to Reviewers'.
- A marked-up copy of your manuscript that highlights changes made to the original version. You should upload this as a separate file labeled 'Revised Manuscript with Track Changes'.
- An unmarked version of your revised paper without tracked changes. You should upload this as a separate file labeled 'Manuscript'.

If you would like to make changes to your financial disclosure, please include your updated statement in your cover letter. Guidelines for resubmitting your figure files are available below the reviewer comments at the end of this letter.

If applicable, we recommend that you deposit your laboratory protocols in protocols.io to enhance the reproducibility of your results. Protocols.io assigns your protocol its own identifier (DOI) so that it can be cited independently in the future. For instructions see: https://journals.plos.org/plosone/s/submission-guidelines#loc-laboratory-protocols. Additionally, PLOS ONE offers an option for publishing peer-reviewed Lab Protocol articles, which describe protocols hosted on protocols.io. Read more information on sharing protocols at https://plos.org/protocols?utm_medium=editorial-email&utm_source=authorletters&utm_campaign=protocols.

As the corresponding author, your ORCID iD is verified in the submission system and will appear in the published article. PLOS supports the use of ORCID, and we encourage all coauthors to register for an ORCID iD and use it as well. Please encourage your coauthors to verify their ORCID iD within the submission system before final acceptance, as unverified ORCID iDs will not appear in the published article. *Only*  the individual author can complete the verification step; PLOS staff *cannot*  verify ORCID iDs on behalf of authors.

We look forward to receiving your revised manuscript.

Kind regards,

Vineet Kumar

Academic Editor

PLOS One

Journal Requirements:

When submitting your revision, we need you to address these additional requirements.

1. Please ensure that your manuscript meets PLOS ONE's style requirements, including those for file naming. The PLOS ONE style templates can be found at

https://journals.plos.org/plosone/s/file?id=wjVg/PLOSOne_formatting_sample_main_body.pdf and https://journals.plos.org/plosone/s/file?id=ba62/PLOSOne_formatting_sample_title_authors_affiliations.pdf

2. You indicated that you had ethical approval for your study. In your Methods section, please ensure you have also stated whether you obtained consent from parents or guardians of the minors included in the study or whether the research ethics committee or IRB specifically waived the need for their consent.

3. In your ethics statement, please ensure you have provided the full name of the ethics committee(s) that approved this study and specified that approval was granted. If more than 5 committees provided approval, it is acceptable to provide a list as a Supporting Information file.”

4. Your ethics statement should only appear in the Methods section of your manuscript. If your ethics statement is written in any section besides the Methods, please delete it from any other section.

5. Please amend your authorship list in your manuscript file to include author Yao Liu, Longqiang CHEN.

6. Please include captions for your Supporting Information files at the end of your manuscript, and update any in-text citations to match accordingly. Please see our Supporting Information guidelines for more information: http://journals.plos.org/plosone/s/supporting-information.

7. We note that there is identifying data in the Supporting Information file <data.csv>. Due to the inclusion of these potentially identifying data, we have removed this file from your file inventory. Prior to sharing human research participant data, authors should consult with an ethics committee to ensure data are shared in accordance with participant consent and all applicable local laws.

Data sharing should never compromise participant privacy. It is therefore not appropriate to publicly share personally identifiable data on human research participants. The following are examples of data that should not be shared:

-Name, initials, physical address

-Ages more specific than whole numbers

-Internet protocol (IP) address

-Specific dates (birth dates, death dates, examination dates, etc.)

-Contact information such as phone number or email address

-Location data

-ID numbers that seem specific (long numbers, include initials, titled “Hospital ID”) rather than random (small numbers in numerical order)

Data that are not directly identifying may also be inappropriate to share, as in combination they can become identifying. For example, data collected from a small group of participants, vulnerable populations, or private groups should not be shared if they involve indirect identifiers (such as sex, ethnicity, location, etc.) that may risk the identification of study participants.

Additional guidance on preparing raw data for publication can be found in our Data Policy (https://journals.plos.org/plosone/s/data-availability#loc-human-research-participant-data-and-other-sensitive-data) and in the following article: http://www.bmj.com/content/340/bmj.c181.long.

Please remove or anonymize all personal information (<specific identifying information in file to be removed>), ensure that the data shared are in accordance with participant consent, and re-upload a fully anonymized data set. Please note that spreadsheet columns with personal information must be removed and not hidden as all hidden columns will appear in the published file.

8. If the reviewer comments include a recommendation to cite specific previously published works, please review and evaluate these publications to determine whether they are relevant and should be cited. There is no requirement to cite these works unless the editor has indicated otherwise.

[Note: HTML markup is below. Please do not edit.]

Reviewers' comments:

Reviewer's Responses to Questions

**Comments to the Author**

1. Is the manuscript technically sound, and do the data support the conclusions?

The manuscript must describe a technically sound piece of scientific research with data that supports the conclusions. Experiments must have been conducted rigorously, with appropriate controls, replication, and sample sizes. The conclusions must be drawn appropriately based on the data presented.

Reviewer #1: Partly

Reviewer #2: Partly

**********

2. Has the statistical analysis been performed appropriately and rigorously?

Reviewer #1: No

Reviewer #2: No

**********

3. Have the authors made all data underlying the findings in their manuscript fully available?

The PLOS Data policy requires authors to make all data underlying the findings described in their manuscript fully available without restriction, with rare exception (please refer to the Data Availability Statement in the manuscript PDF file). The data should be provided as part of the manuscript or its supporting information, or deposited to a public repository. For example, in addition to summary statistics, the data points behind means, medians and variance measures should be available. If there are restrictions on publicly sharing data—e.g. participant privacy or use of data from a third party—those must be specified.

Reviewer #1: No

Reviewer #2: Yes

**********

4. Is the manuscript presented in an intelligible fashion and written in standard English?

PLOS ONE does not copyedit accepted manuscripts, so the language in submitted articles must be clear, correct, and unambiguous. Any typographical or grammatical errors should be corrected at revision, so please note any specific errors here.

Reviewer #1: Yes

Reviewer #2: Yes

**********

5. Review Comments to the Author

Please use the space provided to explain your answers to the questions above. You may also include additional comments for the author, including concerns about dual publication, research ethics, or publication ethics. (Please upload your review as an attachment if it exceeds 20,000 characters)

Reviewer #1: # Review Report

**Manuscript ID:** PONE-D-26-23329

**Current title:** *A Study on the Impact of Resilience Following Failure on Athletes’ Training Commitment and Its Chain-Mediated Mechanism*

**Recommendation:** **Major Revision**

The paper addresses an interesting and relevant issue in sport psychology and has several strengths, including a relatively large sample, a solid theoretical foundation, and an effort to examine subgroup differences. However, several conceptual, methodological, statistical, and reporting concerns currently limit the validity and interpretation of the findings. In particular, the causal language is not appropriate for a cross-sectional self-report design; the mediation model and structural equation model are incompletely reported; several hypotheses are contradicted or unsupported; measurement validation is insufficient; the clustered sampling design is not adequately addressed; and some multi-group significance values appear to be incorrect.

## Comment No. 1: Revise the title and causal language to reflect the cross-sectional design.

The current title contains the terms “impact” and “mechanism,” both of which imply causal relationships and temporal ordering. Because all key variables were measured through a cross-sectional survey, the study can establish associations and model-consistent indirect effects, but it cannot demonstrate that post-failure resilience causes training meaningfulness, that meaningfulness causes value identification, or that the variables necessarily operate in the proposed sequence. The title, abstract, hypotheses, results, discussion, and conclusion should therefore avoid expressions such as “predicts,” “promotes,” “translates into,” and “psychological mechanism.” More appropriate wording would include “is associated with,” “shows an indirect association with,” and “is consistent with the proposed serial model.”

As one possible option, the authors may consider the title: “Post-Failure Resilience and Training Commitment Among Chinese Athletes: A Cross-Sectional Serial Mediation Analysis of Training Meaningfulness and Value Identification.” The manuscript should also undergo a careful English-language revision to improve precision, concision, consistency, and readability.

## Comment No. 2: Clarify the study design and explain how cluster sampling was handled.

At the beginning of the Methods section, the authors should clearly state that the study used a cross-sectional questionnaire design with multistage stratified cluster sampling, confirmatory factor analysis, structural equation modeling, bootstrap mediation analysis, and multi-group SEM. The sampling subsection should report the number of provinces, institutions, teams, and participants within each cluster, explain how participants were selected, and state whether selection probabilities or sampling weights varied.

Because participants were recruited from teams and institutions, the assumption of independent observations may have been violated. Cluster-robust standard errors, complex-sample procedures, or multilevel SEM should be used where possible. If this is not feasible, the resulting dependence should be acknowledged clearly as a limitation.

## Comment No. 3: Provide complete reporting of the mediation and structural equation models.

The manuscript states that the serial indirect effect is significant, but the Results section does not present a complete mediation table. The authors should report the total effect, the direct effect after including the mediators, the specific indirect effects, and the serial indirect effect through training meaningfulness and value identification. Standardized and unstandardized estimates, bootstrap standard errors, 95% bias-corrected confidence intervals, and the proportion of explained variance (R²) should also be provided.

The structural-model fit statistics should be reported separately from the CFA fit statistics and should include χ², degrees of freedom, the exact p-value, χ²/df, CFI, TLI, RMSEA with its 90% confidence interval, and SRMR. The manuscript should clarify whether the direct effect of resilience on training commitment was estimated and whether the model represents full or partial mediation. Figure 3 should be revised accordingly or supported by a complete statistical results table.

## Comment No. 4: Resolve inconsistencies between the hypotheses and the reported findings.

Several hypotheses are contradicted or unsupported and should be reported transparently. H1b predicts that the resilience-to-meaningfulness relationship will be stronger in individual sports, whereas the reported coefficients indicate a stronger relationship in team sports; therefore, H1b is not supported. H1c, H2b, and H3b concern training-stage differences, but no significant differences across training stages are reported; therefore, these hypotheses are also not supported. H4a cannot be evaluated fully until the indirect-effect estimates and their confidence intervals are presented.

The authors should add a concise hypothesis-testing table in the Results section showing each hypothesis, the predicted relationship, the corresponding estimate, the significance result, and whether the hypothesis is supported. Any post hoc interpretation, particularly regarding stronger effects in team sports, should be identified explicitly as exploratory.

## Comment No. 5: Strengthen measurement validation and address common-method bias.

The four focal measures appear to have been adapted or developed specifically for this study. In the Measurement Instruments section, the authors should state whether validated Chinese versions were available, which items were adopted, modified, or newly written, whether reverse-coded items were used, how translation and back-translation were conducted, whether the pilot sample overlapped with the main sample, and whether exploratory factor analysis was performed before CFA.

Cronbach’s alpha, composite reliability, and AVE are not sufficient to establish measurement quality. The authors should also report item-level standardized loadings, factor correlations, Fornell–Larcker results, HTMT ratios, and fit comparisons for one-, two-, and four-factor models. Because all constructs were measured through the same self-report questionnaire at one time point, common-method variance is an important concern. The manuscript should describe the procedural safeguards used and apply an appropriate statistical assessment.

## Comment No. 6: Correct the multi-group SEM reporting and significance values.

The manuscript states that configural and metric invariance were established, but the supporting evidence is not presented. For sport type, gender, and training stage, the authors should report fit indices for configural, metric, and structural invariance models, together with ΔCFI, ΔRMSEA, Δχ², degrees of freedom, and exact p-values.

The reported significance levels in Table 4 also require correction. For one degree of freedom, Δχ² = 5.35 corresponds to approximately p = .021, Δχ² = 9.71 corresponds to approximately p = .0018, and Δχ² = 5.28 corresponds to approximately p = .022. These values should be corrected in Table 4 and in the related narrative. The authors should also clarify whether the subgroup analyses were confirmatory or exploratory and discuss the implications of multiple testing.

## Comment No. 7: Moderate the interpretation, practical recommendations, and conclusion.

The discussion currently presents the proposed sequence as an established psychological process, but cross-sectional observational evidence cannot confirm temporal ordering or causality. The authors should test plausible alternative models, including reverse, parallel, and direct-effect models, and report the comparison in the Results section. A better fit of the proposed model would improve its empirical plausibility, although it would still not establish causality.

The practical recommendations should be reframed as theory-guided suggestions for future intervention research rather than as demonstrated consequences of the present data. A dedicated Limitations subsection should discuss the cross-sectional design, self-report bias, common-method variance, possible cluster dependence, measurement adaptation, limited national generalizability, uncontrolled confounders, unequal subgroup sizes, and the absence of longitudinal, coach-rated, behavioral, or objective training data. The conclusion should be revised to correspond strictly to the observed associations.

**The authors are advised to address the following questions thoroughly and provide clear, logical, and well-supported responses.**

**Q1. Model ordering:** In the Data Analysis Methods subsection, specify the reverse, parallel, and direct-effect models to be tested. In the Results section, add a model-comparison table showing the fit of these alternatives against the proposed serial model. What empirical evidence supports the ordering resilience → training meaningfulness → value identification → training commitment?

**Q2. Cluster sampling:** In the Study Population and Sample Sources subsection, report the exact number of provinces, institutions, teams, and participants within each cluster. In the Data Analysis Methods subsection, explain how within-cluster dependence was handled. If clustering was not considered, re-estimate the model using cluster-robust or multilevel methods, or provide a clear explanation in the Limitations section.

**Q3. Complete SEM reporting:** In the Results section, insert one table for mediation analysis and another for multi-group invariance analysis. These tables should report the direct, total, and specific indirect effects; bootstrap confidence intervals; model-fit statistics; invariance statistics; Δχ² values; and exact p-values. Figure 3 should also be revised to show the direct effect, explained variance, and complete model structure, or it should be accompanied by a detailed statistical table.

Although the manuscript addresses a useful research question and includes a potentially valuable dataset, the current version requires major revision because the analytical reporting is incomplete, several hypotheses are unsupported, measurement and sampling concerns remain unresolved, and the conclusions extend beyond what can be justified by a cross-sectional self-report design. A careful major revision would strengthen the paper’s methodological rigor, interpretive balance, and practical significance. The recommended revisions are also expected to enhance the overall quality of the manuscript and increase its potential impact on future research.

Reviewer #2: The novelty of the proposed serial mediation model should be more clearly established by explicitly comparing it with existing studies on resilience, meaning-making, and self-determination in sports psychology.

The manuscript should provide stronger theoretical justification for the proposed causal sequence among resilience, training meaningfulness, value identification, and training commitment.

The cross-sectional study design limits causal interpretation; this limitation should be discussed more comprehensively, and longitudinal validation should be recommended.

Greater detail is needed regarding the development, adaptation, and validation of the measurement instruments, particularly for the self-developed scales.

The manuscript should include a more comprehensive assessment of common method bias beyond the currently reported analyses.

The rationale for selecting only Chinese athletes should be better justified, and the implications for the generalizability of the findings should be discussed in greater depth.

The measurement invariance analysis should be reported more comprehensively, including detailed fit indices and results for each stage of invariance testing.

Alternative structural models should be tested and compared to demonstrate that the proposed serial mediation model provides the best representation of the data.

The discussion should critically compare the findings with previous international studies rather than primarily emphasizing consistency with the proposed theoretical framework.

The practical implications should be expanded by providing specific recommendations for coaches, sports psychologists, and training organizations based on the reported findings.

The manuscript should discuss additional potential confounding variables, such as competitive level, coaching style, sport experience, and psychological characteristics, which may influence the observed relationships.

Future research directions should be strengthened by proposing longitudinal, experimental, and cross-cultural studies to validate the robustness and applicability of the proposed theoretical model.

**********

6. PLOS authors have the option to publish the peer review history of their article (what does this mean?). If published, this will include your full peer review and any attached files.

If you choose “no”, your identity will remain anonymous but your review may still be made public.

**Do you want your identity to be public for this peer review?**  For information about this choice, including consent withdrawal, please see our Privacy Policy.

Reviewer #1: **Yes:**  Muhammad Saeed Ahmad

Reviewer #2: **Yes:**  VINEET KUMAR

**********

[NOTE: If reviewer comments were submitted as an attachment file, they will be attached to this email and accessible via the submission site. Please log into your account, locate the manuscript record, and check for the action link "View Attachments". If this link does not appear, there are no attachment files.]

To ensure your figures meet our technical requirements, please review our figure guidelines: https://journals.plos.org/plosone/s/figures

You may also use PLOS’s free figure tool, NAAS, to help you prepare publication quality figures: https://journals.plos.org/plosone/s/figures#loc-tools-for-figure-preparation.

NAAS will assess whether your figures meet our technical requirements by comparing each figure against our figure specifications.

Attachment Submitted filename: PONE_D_26_23329_Review_Report.docx

# Author comment: Author response to Decision Letter 1

**DOI:** 10.1371/journal.pone.0359579.r002

28 Aug 2026

Dear Editors and Reviewers,

Thank you very much for handling our manuscript entitled “Post-failure resilience and athletes’ training engagement: the chain-mediating roles of perceived meaning in training and value identification.” We sincerely appreciate the careful evaluation and constructive comments provided by the reviewers. Based on these comments and suggestions, we have carefully revised the manuscript. The point-by-point responses to the reviewers are presented below.

Please let us know if any additional information is needed.

Thank you very much for your time and consideration.

Sincerely yours,

The Authors

Reviewer #1

Dear Reviewer,

We sincerely thank you for your careful reading of our manuscript and for the comprehensive and constructive comments. Your suggestions addressed several important aspects of the study, including the framing of the cross-sectional design, causal language, structural equation and mediation reporting, hypothesis testing, measurement validation, subgroup analyses, and the appropriate interpretation of the findings. We have revised the manuscript extensively in response. The principal revisions include: reframing the title and empirical interpretations in associational terms; clarifying the cross-sectional design and recruitment procedure; expanding the structural equation model and bootstrap effect decomposition; re-examining all hypotheses and multi-group comparisons; strengthening measurement validation through Fornell–Larcker, HTMT, competing measurement models, and common-method-bias assessments; rechecking the multi-group SEM statistics; comparing reverse, parallel, and direct-effect alternative models; moderating the discussion, practical implications, and conclusion; adding a dedicated limitations section; and providing Supplementary Table S1 with item-level standardized factor loadings for all 24 indicators.

Our point-by-point responses are provided below.

Comment No. 1: Revise the title and causal language to reflect the cross-sectional design

The current title contains the terms “impact” and “mechanism,” both of which imply causal relationships and temporal ordering. Because all key variables were measured through a cross-sectional survey, the study can establish associations and model-consistent indirect effects, but it cannot demonstrate that post-failure resilience causes training meaningfulness, that meaningfulness causes value identification, or that the variables necessarily operate in the proposed sequence. The title, abstract, hypotheses, results, discussion, and conclusion should therefore avoid expressions such as “predicts,” “promotes,” “translates into,” and “psychological mechanism.” More appropriate wording would include “is associated with,” “shows an indirect association with,” and “is consistent with the proposed serial model.” As one possible option, the authors may consider the title: “Post-Failure Resilience and Training Commitment Among Chinese Athletes: A Cross-Sectional Serial Mediation Analysis of Training Meaningfulness and Value Identification.” The manuscript should also undergo a careful English-language revision to improve precision, concision, consistency, and readability.

Our Response: Thank you for identifying this important issue. We agree that a cross-sectional self-report design cannot establish temporal precedence or causal relationships. Accordingly, we revised the title to emphasize association rather than causal impact. The revised title is: “Association Between Post-Failure Recovery Ability and Athletes’ Training Engagement: A Serial Mediation Analysis of Training Meaningfulness and Value Identification.”

We also systematically revised the empirical framing in the Abstract, study aims, hypotheses, Results, Discussion, and Conclusion. Expressions that could be read as causal claims were replaced, where they referred to the present data, with associational language such as “is positively associated with,” “indirect association,” “serial indirect effect,” “is consistent with,” and “is linked to.” The Abstract now explicitly states that the study examines the association between post-failure recovery ability and training engagement and the serial indirect association involving training meaningfulness and value identification.

In addition, the Data Analysis subsection and the alternative-model comparison now state explicitly that differences in model fit reflect only the relative compatibility of alternative theoretical structures with the observed covariance pattern and cannot establish the true temporal order or causal direction among the variables. The Conclusion likewise specifies that the significant serial indirect effect represents a statistical indirect association in cross-sectional data rather than an established causal mechanism.

We retained some process-oriented wording in the theoretical-background sections when summarizing the propositions of resilience theory, meaning-making theory, and self-determination theory. These passages describe established theoretical propositions rather than causal conclusions drawn from the present cross-sectional data. We have, however, made the empirical interpretation of our own findings substantially more cautious and associational.

Comment No. 2: Clarify the study design and explain how cluster sampling was handled

At the beginning of the Methods section, the authors should clearly state that the study used a cross-sectional questionnaire design with multistage stratified cluster sampling, confirmatory factor analysis, structural equation modeling, bootstrap mediation analysis, and multi-group SEM. The sampling subsection should report the number of provinces, institutions, teams, and participants within each cluster, explain how participants were selected, and state whether selection probabilities or sampling weights varied. Because participants were recruited from teams and institutions, the assumption of independent observations may have been violated. Cluster-robust standard errors, complex-sample procedures, or multilevel SEM should be used where possible. If this is not feasible, the resulting dependence should be acknowledged clearly as a limitation.

Our Response: Thank you for this important methodological clarification. After re-examining the recruitment procedure, we concluded that describing the design as formal “multistage stratified cluster sampling” would overstate the probability-based nature of the sampling process. We therefore revised Section 3.1 to describe the procedure more accurately as a cross-sectional questionnaire study using multi-region and multi-training-organization recruitment.

The revised manuscript now reports that participants were recruited from 10 provincial-level administrative regions across several geographic areas of China and were mainly drawn from provincial professional teams and university high-performance sport programs. We also clarify that recruitment was not based on a complete probability sampling frame with known selection probabilities. Consequently, individual selection probabilities were not calculated and sampling weights were not applied. We therefore restrict the inferential scope of the study to testing associations and model fit in the present sample rather than estimating nationally representative parameters for Chinese athletes.

We agree that athletes nested within teams and institutions may violate the assumption of complete independence. However, the final anonymized analytic dataset did not retain institution- or team-level cluster identifiers. Because those identifiers cannot be reconstructed reliably after anonymization, we could not perform valid cluster-robust standard-error estimation or multilevel SEM at the revision stage. Rather than reconstructing clusters using unverifiable assumptions, we now state this limitation explicitly in Section 3.3 and clarify that the athlete is the unit of analysis.

For the same reason, exact institution-level and team-level cluster counts cannot be reconstructed reliably from the final analytic file. We have therefore reported the information that can be verified from the study records (including the 10 provincial-level regions and the major organizational sources) and have avoided inventing cluster counts or post hoc allocation figures. Future studies should retain anonymized team/institution cluster codes so that within-cluster dependence can be evaluated using multilevel or cluster-robust procedures.

Comment No. 3: Provide complete reporting of the mediation and structural equation models

The manuscript states that the serial indirect effect is significant, but the Results section does not present a complete mediation table. The authors should report the total effect, the direct effect after including the mediators, the specific indirect effects, and the serial indirect effect through training meaningfulness and value identification. Standardized and unstandardized estimates, bootstrap standard errors, 95% bias-corrected confidence intervals, and the proportion of explained variance (R²) should also be provided. The structural-model fit statistics should be reported separately from the CFA fit statistics and should include χ², degrees of freedom, the exact p-value, χ²/df, CFI, TLI, RMSEA with its 90% confidence interval, and SRMR. The manuscript should clarify whether the direct effect of resilience on training commitment was estimated and whether the model represents full or partial mediation. Figure 3 should be revised accordingly or supported by a complete statistical results table.

Our Response: We appreciate this detailed recommendation. We substantially expanded both the structural model and the mediation reporting.

First, Section 3.3 now specifies a complete structural model in which six structural paths are estimated simultaneously: RES→MEA, RES→VAL, RES→ENG, MEA→VAL, MEA→ENG, and VAL→ENG. This avoids inferring serial mediation solely from the significance of adjacent paths.

Second, Section 4.3 now reports the structural-model fit separately in the structural-model subsection: χ²(246) = 230.546, χ²/df = 0.937, CFI = 1.000, TLI = 1.002, RMSEA = 0.000, 90% CI [0.000, 0.011], and SRMR = 0.021. The model explains 43.6%, 48.3%, and 50.2% of the variance in MEA, VAL, and ENG, respectively (R² = 0.436, 0.483, and 0.502).

Third, we added a complete effect-decomposition table (Table 4), including unstandardized estimates (B), standardized estimates (β), bootstrap standard errors, and bias-corrected 95% confidence intervals for the direct effect, both specific indirect effects, the serial indirect effect, the total indirect effect, and the total effect:

Direct effect RES→ENG: B = 0.138, β = 0.123, Bootstrap SE = 0.050, 95% BC CI [0.042, 0.234].

Specific indirect effect RES→MEA→ENG: B = −0.051, β = −0.046, Bootstrap SE = 0.046, 95% BC CI [−0.144, 0.033].

Specific indirect effect RES→VAL→ENG: B = 0.015, β = 0.013, Bootstrap SE = 0.040, 95% BC CI [−0.068, 0.091].

Serial indirect effect RES→MEA→VAL→ENG: B = 0.350, β = 0.312, Bootstrap SE = 0.046, 95% BC CI [0.266, 0.455].

Total indirect effect: B = 0.313, β = 0.279, Bootstrap SE = 0.049, 95% BC CI [0.224, 0.412].

Total effect: B = 0.451, β = 0.402, Bootstrap SE = 0.045, 95% BC CI [0.368, 0.546].

Because the RES→ENG direct effect remains statistically significant after MEA and VAL are included, the revised manuscript explicitly identifies the pattern as partial rather than full mediation. Figure 3 was also revised to retain the direct path and to display the explained variance of the endogenous constructs so that the graphical presentation is consistent with the statistical model.

Comment No. 4: Resolve inconsistencies between the hypotheses and the reported findings

Several hypotheses are contradicted or unsupported and should be reported transparently. H1b predicts that the resilience-to-meaningfulness relationship will be stronger in individual sports, whereas the reported coefficients indicate a stronger relationship in team sports; therefore, H1b is not supported. H1c, H2b, and H3b concern training-stage differences, but no significant differences across training stages are reported; therefore, these hypotheses are also not supported. H4a cannot be evaluated fully until the indirect-effect estimates and their confidence intervals are presented. The authors should add a concise hypothesis-testing table in the Results section showing each hypothesis, the predicted relationship, the corresponding estimate, the significance result, and whether the hypothesis is supported. Any post hoc interpretation, particularly regarding stronger effects in team sports, should be identified explicitly as exploratory.

Our Response: Thank you for identifying these inconsistencies. We rechecked all eight pre-specified hypotheses and revised Section 4.4 so that supported and unsupported hypotheses are reported explicitly and transparently.

The revised results show that H1a is supported (RES–MEA: β = 0.660, p < .001), H2a is supported (MEA–VAL: β = 0.682, p < .001), H3a is supported (VAL–ENG: β = 0.693, p < .001), and H4a is supported by the significant serial indirect effect (β = 0.312, 95% BC CI [0.266, 0.455]).

In contrast, H1b, H1c, H2b, and H3b are now explicitly reported as unsupported. In particular, H1b predicted a stronger RES→MEA association in individual sports, whereas the point estimates were Team = 0.699 and Individual = 0.636, and the between-group difference was not significant, Δχ²(1) = 0.984, p = .321. We therefore state explicitly that H1b was not supported and that the observed point estimates did not follow the predicted “individual sports stronger” pattern. The three training-stage hypotheses likewise showed no statistically significant between-stage differences and are reported as unsupported.

We also separated confirmatory and exploratory subgroup comparisons. The sport-type comparison for RES→MEA and the three training-stage comparisons correspond to pre-specified hypotheses and are treated as confirmatory. Other sport-type path comparisons and the gender comparisons are treated as exploratory. For these exploratory tests, Holm adjustment was applied to reduce inflation of Type I error. The unadjusted sport-type difference for MEA→VAL was p = .047, but the Holm-adjusted p-value was .093; accordingly, we do not interpret this result as evidence of a stable sport-type moderation effect.

We carefully considered the suggestion to add a separate hypothesis-testing table. To avoid substantial duplication of Table 4 (indirect effects) and Table 5 (multi-group path comparisons), we instead added a concise, integrated hypothesis-status summary at the end of Section 4.4, explicitly identifying H1a, H2a, H3a, and H4a as supported and H1b, H1c, H2b, and H3b as unsupported. We believe this preserves transparency while minimizing redundant presentation.

Comment No. 5: Strengthen measurement validation and address common-method bias

The four focal measures appear to have been adapted or developed specifically for this study. In the Measurement Instruments section, the authors should state whether validated Chinese versions were available, which items were adopted, modified, or newly written, whether reverse-coded items were used, how translation and back-translation were conducted, whether the pilot sample overlapped with the main sample, and whether exploratory factor analysis was performed before CFA. Cronbach’s alpha, composite reliability, and AVE are not sufficient to establish measurement quality. The authors should also report item-level standardized loadings, factor correlations, Fornell–Larcker results, HTMT ratios, and fit comparisons for one-, two-, and four-factor models. Because all constructs were measured through the same self-report questionnaire at one time point, common-method variance is an important concern. The manuscript should describe the procedural safeguards used and apply an appropriate statistical assessment.

Our Response: We thank the reviewer for emphasizing measurement transparency and discriminant validity. We revised Sections 3.2 and 4.2 substantially.

Section 3.2 now distinguishes the measurement origins more clearly. Post-failure recovery ability and training meaningfulness were developed for the competitive-training context on the basis of established theoretical frameworks and relevant prior research; value identification was contextually adapted from relevant motivational and self-concordance frameworks; and training engagement was simplified and adapted from an existing athletic training engagement measure. The revised text describes the overall development procedure as theory definition → item development/contextual adaptation → expert semantic review → athlete feedback → pilot testing → validation in the formal sample.

We also expanded the description of language and content adaptation. Before the formal survey, a pilot study with 128 athletes was used to evaluate clarity, comprehensibility, and fit with the competitive-training context. Translation/back-translation, expert semantic review, and athlete feedback were used to refine wording, and two doctoral-level reviewers with sport-psychology backgrounds independently evaluated semantic consistency and conceptual equivalence.

Because the four constructs and item assignments had been specified a priori on the basis of theory, item-development procedures, expert review, and pilot testing, the main sample was not used to generate a data-driven factor structure through a new EFA. Instead, the pre-specified four-factor measurement model was evaluated directly using CFA. We have clarified this rationale in the revised Methods.

Measurement validation in the formal sample was expanded beyond Cronbach’s alpha, CR, and AVE. The revised manuscript now reports standardized factor loadings, latent-factor correlations, Fornell–Larcker results, HTMT ratios, and competing measurement-model comparisons. All 24 items loaded significantly on their intended factors, with standardized loadings ranging from 0.643 to 0.825. The highest HTMT value was 0.701, and the four-factor model fit substantially better than the three-factor, two-factor, and one-factor alternatives. In addition, Supplementary Table S1 reports the item-level standardized loading for every indicator.

We also strengthened the common-method-bias assessment. Procedurally, the survey used anonymous and voluntary responding, standardized instructions, expert semantic review, athlete feedback, and pilot testing to reduce evaluation apprehension, ambiguity, and systematic response artifacts. Statistically, we conducted Harman’s single-factor test and compared a one-factor CFA model with the hypothesized four-factor measurement model. The first unrotated factor accounted for 39.19% of the variance, and the one-factor CFA fit was markedly poorer than the four-factor model. We therefore conclude cautiously that the available evidence does not indicate that a single common-method factor dominates the covariance structure. Importantly, the revised Methods explicitly note that these procedures do not completely rule out common-method variance.

Comment No. 6: Correct the multi-group SEM reporting and significance values

The manuscript states that configural and metric invariance were established, but the supporting evidence is not presented. For sport type, gender, and training stage, the authors should report fit indices for configural, metric, and structural invariance models, together with ΔCFI, ΔRMSEA, Δχ², degrees of freedom, and exact p-values. The reported significance levels in Table 4 also require correction. For one degree of freedom, Δχ² = 5.35 corresponds to approximately p = .021, Δχ² = 9.71 corresponds to approximately p = .0018, and Δχ² = 5.28 corresponds to approximately p = .022. These values should be corrected in Table 4 and in the related narrative. The authors should also clarify whether the subgroup analyses were confirmatory or exploratory and discuss the implications of multiple testing.

Our Response: We appreciate the reviewer’s careful statistical checking. We re-examined the multi-group SEM analyses and rewrote the reporting rather than carrying forward the previous significance labels.

The revised Methods specify the sequence of multi-group testing as configural invariance → metric invariance → structural-path invariance, with evaluation based on χ², df, Δχ², Δdf, exact p-values, ΔCFI, and ΔRMSEA. We also specify ΔCFI ≤ 0.010 and ΔRMSEA ≤ 0.015 as complementary criteria when evaluating invariance.

The revised Results now report the following model-comparison statistics. For sport type, the configural model yielded χ²(492) = 450.343 and the metric model χ²(512) = 457.699; Δχ²(20) = 7.356, p = .995, ΔCFI = 0.000, ΔRMSEA = 0.000. Constraining the three core structural paths simultaneously produced Δχ²(3) = 5.934, p = .115. For training stage, the metric-invariance comparison was Δχ²(20) = 19.901, p = .464, with ΔCFI = 0.000 and ΔRMSEA = 0.000; the three-path structural constraint yielded Δχ²(3) = 0.367, p = .947. For gender, the metric-invariance comparison was Δχ²(20) = 17.324, p = .632, and the three-path structural constraint yielded Δχ²(3) = 0.568, p = .904.

Table 5 was also reconstructed to report the group-specific standardized coefficients, Δχ² values, and exact p-values for the core path comparisons. For example, the sport-type RES→MEA comparison is Δχ²(1) = 0.984, p = .321, and the training-stage comparisons are p = .862, .868, and .585. The Δχ² values and p-values flagged in the previous version are therefore no longer used in the revised manuscript; the new report reflects the rechecked model comparisons.

Finally, we now distinguish confirmatory from exploratory subgroup analyses and apply Holm adjustment to exploratory multiple comparisons. The revised Results report both the unadjusted exploratory result where relevant and the adjusted conclusion, thereby reducing the risk of overinterpreting isolated nominal p-values.

Comment No. 7: Moderate the interpretation, practical recommendations, and conclusion

The discussion currently presents the proposed sequence as an established psychological process, but cross-sectional observational evidence cannot confirm temporal ordering or causality. The authors should test plausible alternative models, including reverse, parallel, and direct-effect models, and report the comparison in the Results section. A better fit of the proposed model would improve its empirical plausibility, although it would still not establish causality. The practical recommendations should be reframed as theory-guided suggestions for future intervention research rather than as demonstrated consequences of the present data. A dedicated Limitations subsection should discuss the cross-sectional design, self-report bias, common-method variance, possible cluster dependence, measurement adaptation, limited national generalizability, uncontrolled confounders, unequal subgroup sizes, and the absence of longitudinal, coach-rated, behavioral, or objective training data. The conclusion should be revised to correspond strictly to the observed associations.

Our Response: We agree with the reviewer’s concern and revised the Results, Discussion, practical recommendations, Conclusion, and limitations accordingly.

First, Section 3.3 now defines four alternative structural specifications: (1) the proposed serial model RES→MEA→VAL→ENG; (2) a reverse-order model RES→VAL→MEA→ENG; (3) a parallel-mediator model; and (4) a direct-effect model. Section 4.3 reports the comparison. The proposed serial model showed the best fit: χ²(248) = 232.066, χ²/df = 0.936, CFI = 1.000, TLI = 1.002, RMSEA = 0.000, SRMR = 0.022. The reverse-order model showed poorer fit (χ²(248) = 533.452, CFI = 0.969, RMSEA = 0.039, SRMR = 0.081), as did the parallel model (χ²(247) = 401.250, CFI = 0.983, RMSEA = 0.029, SRMR = 0.071) and the direct-effect model (χ²(248) = 506.779, CFI = 0.972, RMSEA = 0.037, SRMR = 0.116). AIC and BIC comparisons were also more favorable for the proposed serial structure.

We explicitly caution that this relative fit advantage increases the empirical plausibility of the theoretical ordering but does not establish temporal precedence or causality. The Discussion was revised to interpret the findings as associations that are theoretically consistent with the proposed framework rather than as a demonstrated psychological process.

Second, the practical recommendations were reframed as theory-guided and testable directions rather than demonstrated intervention effects. Section 6.2 now states at the outset that the “recovery–meaning–value” framework should be regarded as a practice framework worthy of further testing and that its actual intervention effects require longitudinal or experimental validation.

Third, we added a dedicated “Limitations and Future Directions” section. To keep this section proportionate to the article, we focus on the two broad limitations most central to interpretation: the inability of the cross-sectional design to establish temporal or causal ordering, and the reliance on athlete self-report data from a specific competitive-training context, which limits robustness and external applicability. The section also directs future research toward longitudinal/experimental designs, multi-source data, and more fine-grained contextual indicators. Other methodological constraints, including the unavailability of cluster identifiers and the resulting inability to estimate cluster-robust or multilevel models, are disclosed directly in the Methods so that they are transparent to readers.

Finally, the Conclusion was revised to correspond to the observed associations. It now describes the serial effect as a statistically significant indirect association and explicitly states that it cannot be interpreted as an established temporal sequence or causal mechanism.

Additional Question Q1: Model ordering

In the Data Analysis Methods subsection, specify the reverse, parallel, and direct-effect models to be tested. In the Results section, add a model-comparison table showing the fit of these alternatives against the proposed serial model. What empirical evidence supports the ordering resilience → training meaningfulness → value identification → training commitment?

Our Response: We now specify the reverse-order, parallel-mediator, and direct-effect models in Section 3.3 and compare them with the proposed serial model in Section 4.3. The empirical evidence supporting the proposed order is comparative rather than causal: the proposed serial structure shows better fit and lower information criteria than the plausible alternatives. Most notably, the reverse-order model increases χ² from 232.066 to 533.452, while the parallel and direct-effect models also fit less well.

We therefore interpret the evidence as showing that the observed covariance pattern is more compatible with RES→MEA→VAL→ENG than with the tested alternatives. We do not claim that this establishes the true temporal order. The ordering remains theory-driven, grounded in resilience theory, meaning-making theory, and self-determination theory, and requires longitudinal, cross-lagged, or experimental confirmation.

We considered adding a separate model-comparison table. Because the full fit indices and information-criterion differences for all four structural models are reported consecutively in Section 4.3, and because the manuscript already contains several new statistical tables, we retained the comparison in a compact narrative format to avoid redundant presentation while preserving all key fit information.

Additional Question Q2: Cluster sampling

In the Study Population and Sample Sources subsection, report the exact number of provinces, institutions, teams, and participants within each cluster. In the Data Analysis Methods subsection, explain how within-cluster dependence was handled. If clustering was not considered, re-estimate the model using cluster-robust or multilevel methods, or provide a clear explanation in the Limitations section.

Our Response: We revised the sampling description to reflect the actual recruitment process rather than characterizing it as formal probability-based multistage cluster sampling. The manuscript now reports recruitment from 10 provincial-level administrative regions and identifies the main organizational sources. It also explicitly states that selection probabilities and sampling weights were not available.

The final anonymized analytic dataset did not retain institution- and team-level cluster identifiers. Consequently, exact institution/team cluster counts and participant counts per cluster cannot be reconstructed reliably, and cluster-robust or multilevel re-estimation cannot be performed validly after the fact. We chose not to manufacture cluster identifiers or infer cluster assignments from incomplete information. Instead, Section 3.3 transparently states that the athlete is the unit of analysis and that no cluster-robust standard errors or multilevel estimates were applied because the necessary cluster identifiers were unavailable. We also limit generalization accordingly and identify retention of anonymous cluster codes as an important design improvement for future work.

Additional Question Q3: Complete SEM reporting

In the Results section, insert one table for mediation analysis and another for multi-group invariance analysis. These tables should report the direct, total, and specific indirect effects; bootstrap confidence intervals; model-fit statistics; invariance statistics; Δχ² values; and exact p-values. Figure 3 should also be revised to show the direct effect, explained variance, and complete model structure, or it should be accompanied by a detailed statistical table.

Our Response: We added a complete mediation table (Table 4) that reports the direct effect, two specific indirect effects, serial indirect effect, total indirect effect, and total effect, with B, β, Bootstrap SE, and bias-corrected 95% confidence intervals. We also report R² for all three endogenous constructs and revised Figure 3 to display the direct RES→ENG path, the other structural paths, and the explained variance.

For the multi-group analysis, Section 4.4 now reports the configural, metric, and structural-invariance results for sport type, training stage, and gender, including χ², df, Δχ², exact p-values, ΔCFI, and ΔRMSEA. Table 5 then reports group-specific coefficients and the one-degree-of-freedom path-comparison statistics. Because the invariance statistics are already presented consecutively and in full in Section 4.4, while Table 5 provides the path-level comparisons, we did not add a second invariance table that would reproduce the same values. We have nevertheless ensured that the requested statistics are explicitly available in the revised Results.

Closing statement

We again thank the reviewer for the careful and technically detailed evaluation of our manuscript. The revision prompted us to reconsider the evidential limits of the study, improve the completeness of the SEM and indirect-effect reporting, re-examine the hypothesis and subgroup analyses, strengthen measurement validation, and moderate the practical and theoretical interpretation of the findings. We believe that the revised manuscript is substantially clearer, more transparent, and methodologically better balanced as a result of these comments.

We sincerely appreciate the time and expertise invested in helping us improve the manuscript.

Reviewer #2

Dear Reviewer,

Thank you very much for your careful, constructive, and insightful comments on our manuscript. Your suggestions helped us clarify the theoretical contribution of the study, improve the transparency of the measurement and statistical procedures, and further refine the interpretation of the cross-sectional findings, the scope of generalizability, and the practical implications.

In response to your comments, we have substantially revised the manuscript. The major changes include: more clearly articulating the theoretical contribution of the serial mediation model in relation to resilience, meaning-making, and self-determination research; strengthening the theoretical rationale for the proposed variable sequence and adding alternative structural model comparisons; further reducing causal language; expanding the reporting of scale development, adaptation, and pilot testing; strengthening the procedural and statistical assessment of common method bias; clarifying the rationale for studying Chinese competitive athletes and the limits of generalizability; expanding multi-group invariance and structural path comparisons; strengthening comparisons with international literature in the Discussion; making the practical implications more specific; and adding potential confounding factors together with longitudinal, experimental, and cross-cultural directions for future research.

Our point-by-point responses are provided below.

Comment 1

Reviewer comment: The novelty of the serial mediation model should be more clearly articulated by comparing it with existing resilience, meaning-making, and self-determination research in sport psychology.

Our Response: Thank you for this important suggestion. We agree that the original manuscript introduced sport resilience theory, meaning-making theory, and self-determination theory separately, but did not sufficiently clarify the theoretical increment created by integrating these perspectives within one structural framework.

In the revised Introduction, we now distinguish the primary focus of the three relevant research traditions. Sport resilience research has mainly examined athletes’ adaptation and maintenance or restoration of psychological functioning under stress, adversity, and failure. Meaning-making research has focused more on how athletes interpret adversity through narrative, identity integration, and the reconstruction of purpose. Self-determination research has primarily examined how externally structured training goals may become internalized and increasingly aligned with personal values and more autonomous forms of motivation.

On this basis, we now state more explicitly that the contribution of the present study is not to re-establish the importance of resilience, meaning, or value identification in isolation, but to examine post-failure psychological recovery, reconstruction of training meaning, value internalization, and training engagement within a single integrated model. We further evaluate this integration through the serial indirect effect and comparisons with alternative structural models. We believe this revision more clearly positions the study relative to existing, largely separate, lines of research.

Comment 2

Reviewer comment: The theoretical justification for the proposed causal sequence should be strengthened.

Our Response: Thank you for this comment. We have strengthened the theoretical justification for the proposed sequence of post-failure recovery ability → training meaning → value identification → training engagement, while also revising overly causal wording throughout the manuscript.

The revised manuscript explains the sequence by integrating sport resilience theory, meaning-making theory, and self-determination theory. Post-failure recovery ability is conceptualized as a psychological resource associated with restoring emotional and cognitive stability, re-establishing perceived controllability of training goals, and returning attention to learning and improvement after failure. Meaning-making theory suggests that when failure can be integrated into a personally coherent developmental narrative, training may regain purpose and interpretability. Self-determination theory further suggests that goals experienced as meaningful are more likely to become incorporated into the individual’s value system and to be experienced as self-endorsed. Value identification, in turn, is expected to be closely associated with more stable and sustained engagement in training.

Importantly, we did not rely on theoretical reasoning alone to support this ordering. We added comparisons with a reversed mediator order model, a parallel mediation model, and a direct-effect model. The theoretically specified serial model showed better statistical fit. However, we explicitly state that these comparisons indicate only the relative compatibility of alternative theoretical structures with the observed cross-sectional covariance pattern and do not establish temporal precedence or causality.

Comment 3

Reviewer comment: The cross-sectional design limits causal inference, and longitudinal validation is needed.

Our Response: We fully agree and have revised the manuscript accordingly. First, causal expressions in the Results, Discussion, and Conclusion were systematically replaced with more cautious terms such as association, structural relationship, indirect relationship, and possible psychological pathway. Second, in the alternative-model section, we now explicitly state that superior model fit cannot establish the true temporal order or causal direction of the variables. Third, the Conclusion clarifies that the significant serial indirect effect represents a statistical indirect association in cross-sectional data and cannot be interpreted as evidence of temporal or causal mediation. Finally, the Limitations and Future Directions section now explicitly recommends longitudinal designs to examine temporal ordering and experimental or intervention studies to evaluate the malleability of the proposed psychological processes.

Comment 4

Reviewer comment: More detailed information should be provided regarding the development, adaptation, and validation of the measurement instruments, particularly the self-developed measures.

Our Response: Thank you for emphasizing measurement transparency. We substantially expanded the Measures section. The revised manuscript now clearly distinguishes the sources of the four measures: post-failure recovery ability and training meaning were developed for the competitive training context based primarily on established theoretical frameworks and relevant sport research; value identification was contextually adapted from existing theoretical and measurement content; and training engagement was simplified and revised from an existing athletic training engagement scale.

We also describe the overall development procedure as theory definition → item development or contextual adaptation → expert semantic review → athlete feedback → pilot testing → validation in the formal sample. Before the formal survey, a pilot test was conducted with 128 athletes to evaluate comprehensibility, semantic clarity, and contextual relevance. The revision also reports the use of translation/back-translation procedures where applicable, expert semantic review, and athlete feedback. Two doctoral-level reviewers with sport psychology backgrounds independently examined semantic consistency and conceptual equivalence.

In addition, we now report Cronbach’s alpha, composite reliability (CR), average variance extracted (AVE), and standardized factor loadings for the measures, and we further evaluate construct validity using CFA, the Fornell–Larcker criterion, HTMT, and competing measurement models in the formal sample.

Comment 5

Reviewer comment: A more comprehensive assessment of common method bias is recommended beyond the analyses currently reported.

Our Response: Thank you for this recommendation. Because all four focal variables were obtained from athlete self-reports at the same time point, we expanded both the procedural and statistical treatment of potential common method bias.

At the procedural level, the questionnaire was administered anonymously and voluntarily using standardized instructions and procedures to reduce evaluation apprehension and administration-related variation. Before the formal survey, expert semantic review, athlete feedback, and pilot testing were used to reduce ambiguity, conceptual overlap, and misunderstanding in the item wording.

At the statistical level, we report Harman’s single-factor test, in which the first factor accounted for 39.19% of the total variance, and we further compare a one-factor CFA model with the theoretically specified four-factor measurement model. The one-factor model showed substantially poorer fit, indicating that a single latent factor was insufficient to account for the major covariance structure among the items. We also explicitly note in the revised manuscript that these procedures do not completely rule out common method variance; they are used only to assess whether it appears to constitute a dominant systematic threat. We therefore retain appropriate caution in interpreting the findings.

Comment 6

Reviewer comment: The rationale for selecting only Chinese athletes should be better justified, and the implications for the generalizability of the findings should be discussed in greater depth.

Our Response: Thank you for raising this issue. We added relevant clarification in the Introduction, the Sample and Recruitment section, and the Limitations section.

The revised manuscript explains that Chinese competitive athletes typically train within relatively systematic and continuous training, evaluation, and stage-based selection structures, in which performance feedback, within-team competition, and training-goal adjustment are recurring features of athletes’ developmental experience. Thus, failure and negative evaluation provide a meaningful empirical context in which to examine recovery, meaning, and value identification. Importantly, we clarify that selecting Chinese competitive athletes was not intended to imply that the proposed psychological relationships are uniquely “Chinese.” Rather, the Chinese competitive training system is treated as a clearly structured empirical context in which to test the statistical compatibility of the proposed model.

We also clarify that the sample was not obtained through probability sampling from a complete sampling frame with known selection probabilities and therefore is not intended to provide nationally representative parameter estimates for Chinese athletes. In the Limitations section, we further note that training organization, coach–athlete interaction, evaluation practices, and sport culture may differ across countries and regions. Accordingly, the present findings should not be generalized directly to other cultural or training-system contexts without further validation. We therefore recommend replication and cross-cultural comparison in future research.

Comment 7

Reviewer comment: Measurement invariance should be examined more comprehensively, with detailed fit indices reported at each stage.

Our Response: Thank you for this suggestion. We expanded the multi-group analysis and now report the fit of each relevant stage in detail. For sport type, training stage, and gender, we first tested configural invariance and then metric invariance by constraining corresponding factor loadings to equality across groups. After metric invariance was supported, we tested the equality of the core structural paths across groups.

For each stage, the revised manuscript reports chi-square, degrees of freedom, CFI, TLI, RMSEA, and SRMR, and the nested-model comparisons further report Δchi-square, Δdf, exact p values, ΔCFI, and ΔRMSEA.

We also clarify the scope of the invariance analysis. The purpose of the multi-group analyses was to compare structural path coefficients rather than latent means. Therefore, structural path comparisons were conducted after configural and metric invariance were supported. Because the study did not compare latent means, scalar invariance was not treated as a necessary prerequisite for the focal structural-path comparisons. The revised analyses indicate acceptable configural and metric invariance across sport type, training stage, and gender, and the core structural relations were generally stable across groups.

Comment 8

Reviewer comment: Alternative structural models should be tested and compared.

Our Response: Thank you for this suggestion. We added a systematic comparison of alternative structural models while keeping the same four-factor measurement structure. Specifically, we compared: (a) the theoretically constrained serial model, RES → MEA → VAL → ENG, with the RES → ENG direct path retained; (b) a reversed-order model, RES → VAL → MEA → ENG; (c) a parallel mediation model in which MEA and VAL operate in parallel; and (d) a direct-effect model without the serial mediation process.

The theoretically specified serial model showed the strongest overall statistical fit. The reversed-order, parallel, and direct-effect models all showed weaker fit to varying degrees. However, we explicitly emphasize that this result means only that the proposed serial structure is more compatible with the observed cross-sectional covariance pattern than the tested alternatives. It does not establish the actual temporal sequence between MEA and VAL or any causal direction.

Comment 9

Reviewer comment: The Discussion should critically compare the findings with previous international studies rather than simply discussing theoretical consistency.

Our Response: Thank you for this valuable comment. We substantially expanded Sections 5.1–5.4 so that the Discussion now compares the findings more directly with previous international research rather than relying mainly on theoretical explanation.

In Section 5.1, the positive association between post-failure recovery ability and training meaning is compared with international resilience research by Galli and Vealey, Fletcher and Sarkar, and Sarkar and Fletcher, together with work by Carless and Douglas and Oblinger-Peters and colleagues on meaning and narrative in sport. We now emphasize that the present study provides a more direct empirical link between recovery-oriented adaptation and the meaning assigned to continued training.

In Section 5.2, we compare the association between training meaning and value identification with international work on meaning, identity integration, and internalization. We also emphasize that training meaning and value identification are closely related but empirically distinguishable psychological levels rather than interchangeable constructs.

In Section 5.3, we compare the value identification–engagement association with self-determination and training-engagement research. A notable pattern in the full model is that VAL → ENG is strong, whereas the direct MEA → ENG path is not significant once value identification is included. We therefore discuss the possibility that simply understanding why training matters may be insufficient for higher engagement unless this meaning becomes more deeply connected with the athlete’s self-value system.

Finally, in Section 5.4, we compare the non-significant moderation results for sport type, training stage, and gender with international literature emphasizing contextual and developmental differences. We distinguish differences in the levels of psychological experiences between groups from differences in the structural relations among psychological variables. We believe these revisions make the Discussion more comparative, critical, and internationally situated.

Comment 10

Reviewer comment: The practical implications should be expanded with more specific recommendations for coaches, sport psychologists, and training organizations.

Our Response: Thank you for this suggestion. We expanded and operationalized the practical implications around a “recovery–meaning–value” framework. The revised manuscript now provides five concrete directions: establishing a learning-oriented language for reviewing failure in routine training communication; using post-failure recovery ability as an auxiliary process indicator rather than a performance criterion; creating cumulative evidence and narratives that help athletes maintain training meaning after setbacks; strengthening value alignment through participatory goal setting, role negotiation, and communication; and adapting a common support framework to specific training contexts rather than assuming fixed group-specific interventions.

We also make the responsible actors more explicit. Coaches can focus on adjusting failure-related feedback and daily training communication; sport psychology practitioners can support psychological re-stabilization, meaning reconstruction, and value clarification after setbacks; and training organizations can provide institutional support through evaluation rules, feedback procedures, and support mechanisms. Because the present study is cross-sectional, we also state that these recommendations are theory- and association-informed and that their intervention effects require longitudinal or experimental validation.

Comment 11

Reviewer comment: Potential confounding factors, including competitive level, coaching style, sport experience, and individual psychological characteristics, should be discussed.

Our Response: Thank you for this important point. We added this issue to the Limitations and Future Directions section. The revised manuscript notes that although competitive level and training years were recorded and described, the current structural model did not systematically include competitive level, training duration, coach interaction or coaching style, or individual psychological characteristics as covariates or contextual factors. These variables may be associated with multiple focal constructs simultaneously.

Accordingly, we now state that the robustness of the observed structural associations after accounting for a broader range of contextual and individual differences remains to be examined. Future studies may incorporate such variables as covariates, contextual factors, or potential boundary conditions in longitudinal, multilevel, or more complex structural models.

Comment 12

Reviewer comment: Future research directions should include longitudinal, experimental, and cross-cultural studies.

Our Response: Thank you for this suggestion. We have expanded the Future Directions section to make these three research directions explicit and to clarify what each design can contribute. We now recommend longitudinal studies to evaluate the temporal relationships among post-failure recovery ability, training meaning, value identification, and training engagement; experimental or intervention studies to examine the malleability of post-failure recovery, meaning reconstruction, and value clarification processes; and replication and cross-cultural comparison across athlete samples from different countries and cultural contexts to assess cross-cultural stability, potential boundary conditions, and the broader applicability of the model. We believe these approaches will help distinguish more clearly among statistical association, temporal ordering, and potential causal mechanisms.

Once again, we sincerely thank the reviewer for these thoughtful and constructive comments. The revisions have helped us improve the manuscript in terms of theoretical positioning, measurement transparency, statistical completeness, interpretive caution, and the specificity of the practical and future research implications. We believe that the revised manuscript now addresses the reviewer’s major concerns more fully and has been substantially strengthened as a result.

Closing

We sincerely thank the editors and reviewers again for their careful evaluation and constructive suggestions. We believe that the revisions have substantially improved the clarity, methodological transparency, and overall quality of the manuscript.

Attachment Submitted filename: Response to Reviewers.docx

# Peer review: Decision Letter 1

**DOI:** 10.1371/journal.pone.0359579.r003

**Authors:** Vineet Kumar

15 Sep 2026

<p>Post-failure resilience and athletes’ training engagement: the chain-mediating roles of perceived meaning in training and value identification

PONE-D-26-23329R1

Dear Dr. Liu,

We’re pleased to inform you that your manuscript has been judged scientifically suitable for publication and will be formally accepted for publication once it meets all outstanding technical requirements.

Within one week, you’ll receive an e-mail detailing the required amendments. When these have been addressed, you’ll receive a formal acceptance letter and your manuscript will be scheduled for publication.

An invoice will be generated when your article is formally accepted. Please note, if your institution has a publishing partnership with PLOS and your article meets the relevant criteria, all or part of your publication costs will be covered. Please make sure your user information is up-to-date by logging into Editorial Manager at Editorial Manager® and clicking the ‘Update My Information' link at the top of the page. For questions related to billing, please contact billing support.

If your institution or institutions have a press office, please notify them about your upcoming paper to help maximize its impact. If they’ll be preparing press materials, please inform our press team as soon as possible -- no later than 48 hours after receiving the formal acceptance. Your manuscript will remain under strict press embargo until 2 pm Eastern Time on the date of publication. For more information, please contact onepress@plos.org.

Kind regards,

Vineet Kumar

Academic Editor

PLOS One

Additional Editor Comments (optional):

The authors have adequately addressed all the reviewers comments and concerns. The revised manuscript is now suitable for publication.

Reviewers' comments:

Reviewer's Responses to Questions

**Comments to the Author**

1. If the authors have adequately addressed your comments raised in a previous round of review and you feel that this manuscript is now acceptable for publication, you may indicate that here to bypass the “Comments to the Author” section, enter your conflict of interest statement in the “Confidential to Editor” section, and submit your "Accept" recommendation.

Reviewer #1: All comments have been addressed

Reviewer #2: All comments have been addressed

**********

2. Is the manuscript technically sound, and do the data support the conclusions?

The manuscript must describe a technically sound piece of scientific research with data that supports the conclusions. Experiments must have been conducted rigorously, with appropriate controls, replication, and sample sizes. The conclusions must be drawn appropriately based on the data presented.

Reviewer #1: Yes

Reviewer #2: Yes

**********

3. Has the statistical analysis been performed appropriately and rigorously?

Reviewer #1: Yes

Reviewer #2: Yes

**********

4. Have the authors made all data underlying the findings in their manuscript fully available?

The PLOS Data policy requires authors to make all data underlying the findings described in their manuscript fully available without restriction, with rare exception (please refer to the Data Availability Statement in the manuscript PDF file). The data should be provided as part of the manuscript or its supporting information, or deposited to a public repository. For example, in addition to summary statistics, the data points behind means, medians and variance measures should be available. If there are restrictions on publicly sharing data—e.g. participant privacy or use of data from a third party—those must be specified.

Reviewer #1: Yes

Reviewer #2: Yes

**********

5. Is the manuscript presented in an intelligible fashion and written in standard English?

PLOS ONE does not copyedit accepted manuscripts, so the language in submitted articles must be clear, correct, and unambiguous. Any typographical or grammatical errors should be corrected at revision, so please note any specific errors here.

Reviewer #1: Yes

Reviewer #2: Yes

**********

6. Review Comments to the Author

Please use the space provided to explain your answers to the questions above. You may also include additional comments for the author, including concerns about dual publication, research ethics, or publication ethics. (Please upload your review as an attachment if it exceeds 20,000 characters)

Reviewer #1: I have carefully reviewed the revised manuscript and the authors’ point-by-point responses. The authors have satisfactorily addressed the major methodological, statistical, and interpretive concerns raised in the previous review. The revised manuscript now reflects the cross-sectional design appropriately, avoids unsupported causal claims, provides more complete SEM and mediation reporting, strengthens measurement validation and common-method-bias assessment, and improves the subgroup and invariance analyses. The limitations related to recruitment, clustering, self-report data, and generalizability are now clearly acknowledged. The conclusions are appropriately framed as associations and are supported by the reported data. I found no additional research-ethics or publication-ethics concerns in the submitted materials. Overall, the manuscript has been substantially improved, and I have no further substantive comments. I recommend acceptance for publication in PLOS ONE.

Reviewer #2: Authors have did well to address the comments raised in earlier version. I am glad to recommend this paper for publication.

**********

7. PLOS authors have the option to publish the peer review history of their article (what does this mean?). If published, this will include your full peer review and any attached files.

If you choose “no”, your identity will remain anonymous but your review may still be made public.

**Do you want your identity to be public for this peer review?**  For information about this choice, including consent withdrawal, please see our Privacy Policy.

Reviewer #1: **Yes:**  Muhammad Saeed Ahmad

Reviewer #2: **Yes:**  Vineet Kumar

**********

Attachment Submitted filename: Reviewer1_Second_Round_Report.docx

# Editor report: Acceptance letter

**DOI:** 10.1371/journal.pone.0359579.r004

**Authors:** Vineet Kumar

PONE-D-26-23329R1

PLOS One

Dear Dr. Liu,

I'm pleased to inform you that your manuscript has been deemed suitable for publication in PLOS One. Congratulations! Your manuscript is now being handed over to our production team.

At this stage, our production department will prepare your paper for publication. This includes ensuring the following:

* All references, tables, and figures are properly cited

* All relevant supporting information is included in the manuscript submission,

* There are no issues that prevent the paper from being properly typeset

You will receive further instructions from the production team, including instructions on how to review your proof when it is ready. Please keep in mind that we are working through a large volume of accepted articles, so please give us a few days to review your paper and let you know the next and final steps.

Lastly, if your institution or institutions have a press office, please let them know about your upcoming paper now to help maximize its impact. If they'll be preparing press materials, please inform our press team within the next 48 hours. Your manuscript will remain under strict press embargo until 2 pm Eastern Time on the date of publication. For more information, please contact onepress@plos.org.

You will receive an invoice from PLOS for your publication fee after your manuscript has reached the completed accept phase. If you receive an email requesting payment before acceptance or for any other service, this may be a phishing scheme. Learn how to identify phishing emails and protect your accounts at https://explore.plos.org/phishing.

If we can help with anything else, please email us at customercare@plos.org.

Thank you for submitting your work to PLOS One and supporting open access.

Kind regards,

PLOS One Editorial Office Staff

on behalf of

Dr. Vineet Kumar

Academic Editor

PLOS One
